# Distinct Pathways Mediate the Sorting of Tail-Anchored Proteins to the Plastid Outer Envelope

**DOI:** 10.1371/journal.pone.0010098

**Published:** 2010-04-14

**Authors:** Preetinder K. Dhanoa, Lynn G. L. Richardson, Matthew D. Smith, Satinder K. Gidda, Matthew P. A. Henderson, David W. Andrews, Robert T. Mullen

**Affiliations:** 1 Department of Molecular and Cellular Biology, University of Guelph, Guelph, Ontario, Canada; 2 Department of Biology, Wilfrid Laurier University, Waterloo, Ontario, Canada; 3 Department of Biochemistry and Biomedical Sciences, McMaster University, Hamilton, Ontario, Canada; University of Massachusetts Amherst, United States of America

## Abstract

**Background:**

Tail-anchored (TA) proteins are a distinct class of membrane proteins that are sorted post-translationally to various organelles and function in a number of important cellular processes, including redox reactions, vesicular trafficking and protein translocation. While the molecular targeting signals and pathways responsible for sorting TA proteins to their correct intracellular destinations in yeasts and mammals have begun to be characterized, relatively little is known about TA protein biogenesis in plant cells, especially for those sorted to the plastid outer envelope.

**Methodology/Principal Findings:**

Here we investigated the biogenesis of three plastid TA proteins, including the 33-kDa and 34-kDa GTPases of the translocon at the outer envelope of chloroplasts (Toc33 and Toc34) and a novel 9-kDa protein of unknown function that we define here as an outer envelope TA protein (OEP9). Using a combination of *in vivo* and *in vitro* assays we show that OEP9 utilizes a different sorting pathway than that used by Toc33 and Toc34. For instance, while all three TA proteins interact with the cytosolic OEP chaperone/receptor, AKR2A, the plastid targeting information within OEP9 is distinct from that within Toc33 and Toc34. Toc33 and Toc34 also appear to differ from OEP9 in that their insertion is dependent on themselves and the unique lipid composition of the plastid outer envelope. By contrast, the insertion of OEP9 into the plastid outer envelope occurs in a proteinaceous-dependent, but Toc33/34-independent manner and membrane lipids appear to serve primarily to facilitate normal thermodynamic integration of this TA protein.

**Conclusions/Significance:**

Collectively, the results provide evidence in support of at least two sorting pathways for plastid TA outer envelope proteins and shed light on not only the complex diversity of pathways involved in the targeting and insertion of proteins into plastids, but also the molecular mechanisms that underlie the delivery of TA proteins to their proper intracellular locations in general.

## Introduction

Tail-anchored (TA) proteins are a unique class of integral membrane proteins that possess a cytosolic N-terminal functional domain, followed by a single transmembrane domain (TMD) near or at their C terminus, and a short C-terminal hydrophilic tail [1]. TA proteins are also unique because, unlike the classical type II membrane protein family that possess the same topology (i.e., N_out_-C_in_), their C-terminal TMD emerges from the ribosome only after the termination of translation and, thus, their sorting from the cytosol to the proper organelle membrane occurs *a priori* in a post-translational manner.

TA proteins are associated with all intracellular membranes and participate in a remarkably wide array of physiologically important processes. Consequently, a considerable amount of research has focused in the past few years on understanding their biogenesis, particularly the molecular mechanisms underlying their targeting to and insertion into specific membranes in yeasts and mammals [2]. For instance, the targeting information in almost all TA proteins in these organisms has been demonstrated to be located within their C-terminal TMDs and flanking sequences. Furthermore, the functional nature of these C-terminal targeting signals with regards to their membrane selectivity have been shown to be conveyed, not by primary sequence motifs, but, rather, by distinct physico-chemical properties, such as their net charge and/or the overall hydrophobicity of the TMD.

In terms of the machinery that mediate the targeting and/or insertion of TA proteins to their specific intracellular destinations, several of these have been recently characterized, again primarily in yeasts and mammals, and, with the exception of peroxisome-destined TA proteins [3], [4], most TA proteins in these organisms utilize novel organelle import pathways that do not overlap with those used by their non-TA membrane protein counterparts. Mitochondrial TA proteins, for instance, bypass the translocase of the outer mitochondrial membrane (TOM complex) and utilize instead the mitochondrial sorting and assembly machinery (SAM) [5], and/or the unique lipid composition of the mitochondrial outer membrane [6], [7] in order to ensure their selective targeting. Likewise, the targeting and insertion of ER-destined TA proteins appears to be distinct from the classical signal recognition particle (SRP)/Sec61 co-translational/translocation pathway used by most other ER membrane proteins. ER-destined TA proteins rely instead on several alternative and possibly parallel pathways, including the GET complex [8], [9], Hsp40/Hsc70 [10], [11] or, at least in mammals, other cytosolic chaperones [12] and the unique lipid composition of the ER membrane [13], [14]. How other TA proteins in yeast and mammals or those in other evolutionarily diverse organisms are selectively targeted to their proper intracellular destinations, remain important unanswered questions.

In plants, our understanding of TA protein biogenesis is rudimentary because only a few authentic plant TA proteins have been identified and characterized in terms of their targeting and/or membrane insertion. These include the peroxisomal isoform of ascorbate peroxidase [15], [16], the ER, mitochondrial and/or plastidial isoforms of cytochrome *b*_5_ (Cb5) [17]–[19], certain members of the SNARE protein family [20], the 34-kDa receptor subunit of the translocon at the outer envelope of chloroplasts from pea (*Pisum sativum*; psToc34) [21]–[24], and its homologs in *Arabidopsis thaliana*, Toc33 and Toc34 [25]–[27].

*Arabidopsis* Toc33 and Toc34 function as related, but distinct, substrate-specific GTPase receptors and/or regulators involved in plastid protein import [28]. However, while Toc33 (the presumed ortholog of psToc34; [29]) and Toc34 have been examined with respect to their targeting and membrane insertion, the majority of these studies, as well as those involving psToc34, have yielded conflicting results in regards to the nature of their targeting information [30]. Likewise, it is unclear whether the insertion of Toc33 and Toc34 is mediated by distinct proteinaceous and/or membrane lipid factors, analogous to the unique insertion of ER- and mitochondrial-localized TA proteins, and whether these factors are utilized also by other TA and/or non-TA outer envelope proteins (OEPs) [30], [31]. Thus, elucidating the means by which other plastid TA proteins are targeted to and inserted into the outer envelope is important, not only in terms of their comparative sorting to Toc33 and Toc34, but also for our overall understanding of the sorting of OEPs in general.

Towards this end, we describe here the results of a comprehensive series of *in vivo* and *in vitro* experiments aimed at characterizing and comparing the biogenesis of three *Arabidopsis* TA OEPs: Toc33, Toc34, and a novel 9-kDa putative TA protein (named here ‘OEP9’) that is of unknown function and was recently identified in bioinformatics-based screen for *Arabidopsis* TA proteins [32]. Overall, we demonstrate that OEP9 is a *bona fide* TA plastid outer envelope protein and that, like other OEPs, including Toc33 and Toc34, it relies on the ankryin repeat-containing protein, AKR2A, as a chaperone/receptor for its initial targeting from the cytosol to plastids. OEP9 is distinct from Toc33 and Toc34, however, with regards to the nature of its molecular targeting signal and the membrane protein and lipid components involved in its insertion into the chloroplast outer envelope. The implications of these results in terms of the diversity of OEP sorting pathways, including those responsible for TA OEPs, and the membrane specificity of TA protein targeting in plant cells in general are discussed.

## Results

### Protein sequence features of *Arabidopsis* OEP9, (co)expression profiling and evolutionary analysis of predicted OEP9 homologues in other plant species

**Figure 1:**
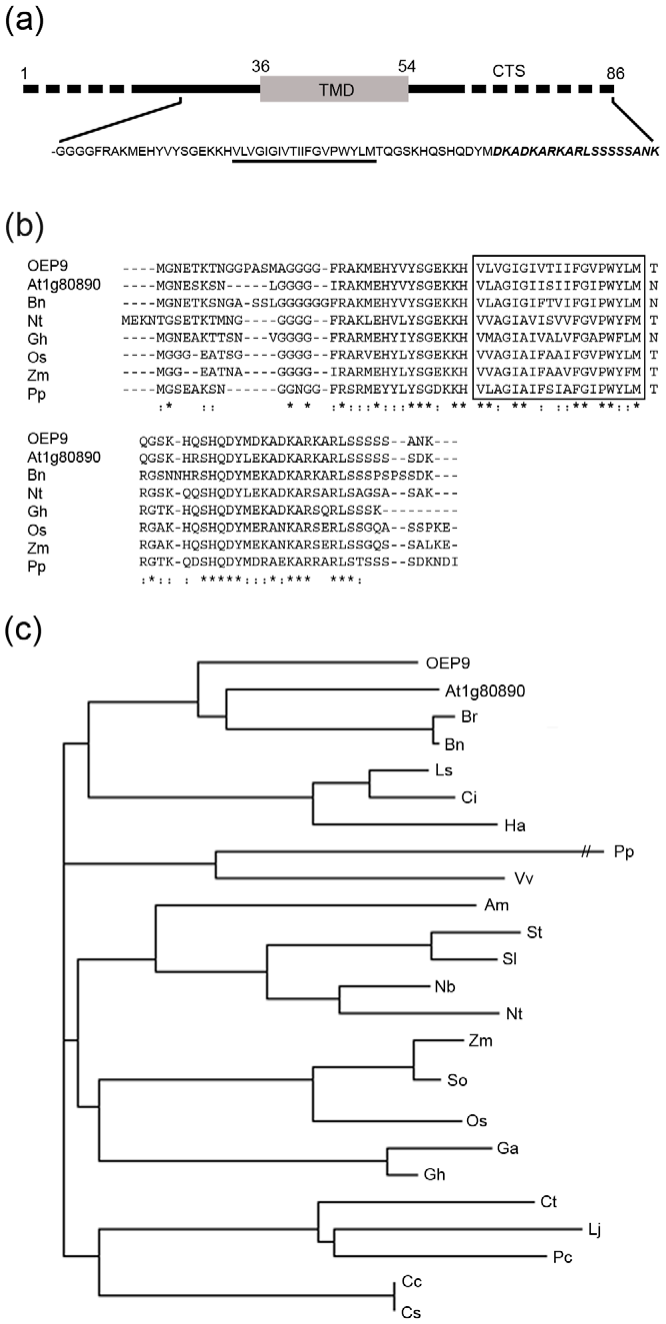
Schematic representation of *Arabidopsis* OEP9 and amino acid alignment and phylogenetic tree of OEP9 and predicted homologues. (a) Schematic illustration of OEP9. Numbers denote the relative position of specific amino acid residues including those that delineate the protein's single putative TMD (shaded grey, residues 36–54) and hydrophilic C-terminal sequence (CTS) (residues 55–86). Putative intrinsically disordered segments (residues 1–18 and 61–86) are indicated with stippled lines. Shown also is the deduced polypeptide sequence of OEP9's ‘NTC’ domain, including the 20 amino acid residues immediately upstream (N terminal) of the TMD, putative TMD (underlined), and CTS. Italicized and bolded amino acid residues in the CTS are those that are immunorecognized by a polyclonal antibody raised against this (synthetic) peptide sequence (see ‘Materials and Methods’ for details). (b) Multiple sequence alignment of the deduced amino acid sequences of OEP9 (At1g16000) (NCBI Accession No. NP_563987) and predicted homologues from *Arabidopsis* (At1g80890), *Brassica* (Bn), tobacco (Nt), cotton (Gh), rice (Os), maize (Zm) and moss (Pp). Identical amino acids in each protein are indicated by asterisks, and strongly similar residues are indicated by colons. Boxed are the single putative TMD in these proteins. (c) Dendogram showing the evolutionary relationship of OEP9, At1g80890 and predicted (protein) homologues from *Brassica rapa* (Br), *Brassica napus* (Bn), *Lactuca saligna* (Ls), *Cichorium intybus* (Ci), *Helianthus annuus* (Ha), *Physcomitrella patens* (Pp), *Vitis vinifera* (Vv), *Antirrhinum majus* (Am), *Solanum tuberosum* (St); *Solanum lycoersicum* (Sl), *Nicotiana benthamiana* (Nb), *Nicotiana tabacum* (Nt), *Zea mays* (Zm), *Saccharum officinarum* (So), *Oryza sativa* (Os), *Gossypium arboreum* (Ga), *Gossypium hirsutum* (Gh), *Cyamopsis tetragonoloba* (Ct), *Lotus japonicus* (Lj), *Phaseolus coccineus* (Pc), *Citrus clementina* (Cc), and *Citrus sinensis* (Cs). The branch lengths of the tree are proportional to the divergence.

As illustrated in Figure 1a, OEP9 (At1g16000) is an 86 amino-acid-long putative TA protein, possessing of a single predicted TMD and a 32 amino-acid-long hydrophilic C-terminal sequence (CTS). According to the information provided for the OEP9 gene locus at GenBank and TAIR, the deduced protein is annotated to be of unknown function and possess no putative targeting signal motifs. Moreover, with the exception of its single, α-helix-forming TMD and two predicted intrinsically disordered (unstructured) segments located at its N and C termini (Figure 1a), OEP9 is devoid of any obvious structural and/or functional domains.

Analyses of various *Arabidopsis* tissue expression databases and co-expression mining algorithms, however, revealed that OEP9 expression is relatively high in roots (Figure S1a–c) and is co-regulated with several other genes, most of which encode cytosolic or plastid ribosomal proteins (Figure S1d). These observations suggest OEP9 functions in plastid ribosome biosynthesis in root cells and provide a reasonable explanation for why OEP9 is absent in publicly available proteomic databases of *Arabidopsis* chloroplast envelope membranes (e.g., PPBD [33]), since these studies were conducted with photosynthetic chloroplasts, which are distinct from root plastids in terms of their proteome composition [34], [35].

Web-based searches using BLASTp revealed that putative homologues with an overall high degree of amino acid sequence identity to OEP9 and, likewise, annotated to be of unknown function, exist in *Arabidopsis* (i.e., At1g80890) and several other plant species, including dicots (*Brassica*, tobacco and cotton), monocots (rice and maize), and moss (*Physcomitrella*) (Figure 1b). In addition, phylogentic analysis of these and other putative OEP9 homologues from diverse plant species (Figure 1c) revealed that they are all closely related and that, since no homologues of OEP9 appear to exist in non-plant organisms, such as yeasts, insects or mammals, they likely share a common ancestor that arose before the evolutionary split between vascular (dicots and monocots) and non-vascular (moss) land plants.

### OEP9, Toc33 and Toc34 are localized to the plastid outer envelope in BY-2 cells

**Figure 2:**
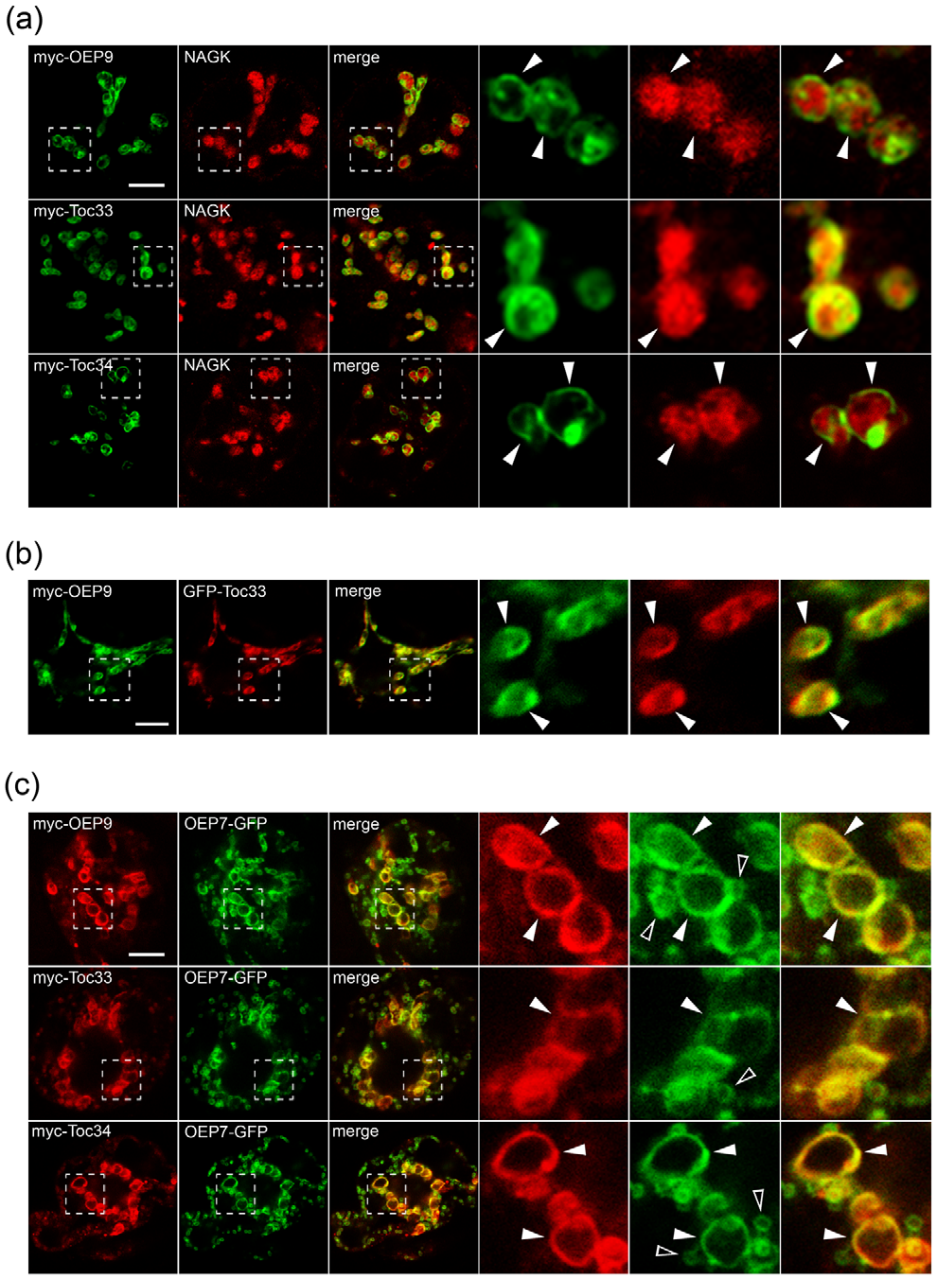
Localization of OEP9, Toc33 and Toc34 in BY-2 cells. (a) Immunofluorescence CLSM micrographs of cells transformed with either (a) myc-OEP9, myc-Toc33 or myc-Toc34 or co-transformed with either (b) myc-OEP9 and GFP-Toc33 or (c) or myc-OEP9, myc-Toc33 or myc-Toc34 and OEP7-GFP. Hatched boxes represent the portion of the cells shown at higher magnification in the panels to the right. Solid arrowheads indicate examples of (a) toridal structures containing myc-tagged OEP9, Toc33 or Toc34 enclosing a spherical structure containing NAGK or larger torus structures containing (b) myc-OEP9 and GFP-Toc33 or (c) myc-tagged OEP9, Toc33, Toc34 and OEP7-GFP. Open arrowheads in (c) indicate examples of the smaller torus structures containing OEP7-GFP, but not myc-tagged OEP9, Toc33 or Toc34. Bars = 10 µm.

Although OEP9 was predicted by Kriechbaumer et al [32] to be localized to mitochondria, this was not confirmed experimentally and it contradicted our results that this protein localizes specifically to plastids. As shown in Figure 2a, transient expression of N-terminal myc-epitope-tagged OEP9 (myc-OEP9) in tobacco BY-2 suspension cells, followed by indirect immunofluorescence confocal laser-scanning microscopy, revealed that the protein localized to numerous toroidal-shaped fluorescent structures that enclosed the punctate/spherical fluorescent structures containing the endogenous plastid stromal protein N-acetylglutamate kinase (NAGK) [36]. These results are consistent with OEP9 being localized to the outer envelope of plastids in these cells, i.e., undifferentiated heterotrophic plastids [37]. Indeed, similar localization results were observed for N-terminal myc-tagged versions of Toc33 (myc-Toc33) and Toc34 (myc-Toc34) (Figure 2a). Control experiments including mock transformations with empty plasmid vector alone or omission of anti-myc IgGs during immunofluorescence staining of BY-2 cells transformed with myc-OEP9 yield no (epi)fluorescence, as expected (Figure S2a).

We showed also that either co-expression of myc-OEP9 and GFP-Toc33 (a chimera consisting of the green fluorescent protein [GFP] appended at its C terminus to Toc33) (Figure 2b) or co-expression of myc-OEP9, myc-Toc33 or myc-Toc34 and OEP7-GFP (consisting of the 7-kDa *Arabidopsis* non-TA OEP appended at its C terminus to GFP [38]) (Figure 2c), resulted in all of these proteins co-localized in the plastid outer envelope in BY-2 cells. Similar results were observed for co-expressed myc-OEP9 and OEP7-GFP in transformed *Arabidopsis* suspension cells (Figure S2b) and co-expressed GFP-OEP9 (consisting of GFP appended at its C terminus to OEP9) and Tic40-RFP (consisting of the 40 kDa subunit of the translocon at the inner envelope of chloroplasts [Tic40; Ref. 39] fused to the red fluorescent protein [RFP]) in transformed *Arabidopsis* leaf epidermal cells (Figure S2c). Moreover, we showed that co-expressed OEP9 lacking an N-terminal myc epitope tag and myc-Toc33 colocalized in BY-2 cells (Figure S2d). OEP9 being immunodetected in these latter cells using polyclonal antibodies raised against a synthetic peptide corresponding to an amino acid sequence in the protein's CTS (refer to Figure 1a); however, due limited availability of this antibody reagent, mostly myc-tagged versions of OEP9 were employed in the remainder of the experiments described in this study. Nonetheless, these results with OEP9 and those presented also in Figures 2 and S2 confirm that the sorting of (myc-)OEP9 in BY-2 cells, serving as a well-established *in vivo* targeting system for ectopically-expressed proteins [40], faithfully reflects its localization in *Arabidopsis* cells.

Notably, OEP7-GFP in (co)transformed cells localized also to several, relatively smaller torus structures that were devoid of myc-tagged OEP9, Toc33 or Toc34 (Figures 2c and S1b) or did not delineate the punctate/spherical fluorescent structures attributable to endogenous stromal NAGK (Figure S3). These smaller OEP7-GFP-containing torus structures did, however, delineate the punctate/spherical structures attributable to endogenous E1β (Figure S3), a protein subunit of the pyruvate dehydrogenase complex located in the mitochondrial matrix [41]. These results indicate that OEP7-GFP, a commonly used marker (fusion) protein for the chloroplast outer envelope in other studies involving transiently-transformed *Arabidopsis* leaf cell protoplasts [38], [42], [43], sorts also to the mitochondrial outer membrane in suspension cells, perhaps as a consequence of its ectopic (over)expression or a (cryptic) mitochondrial targeting signal that functions depending on the cell type and/or cell function.

### Membrane topology and insertion of OEP9, Toc33 and Toc34

**Figure 3:**
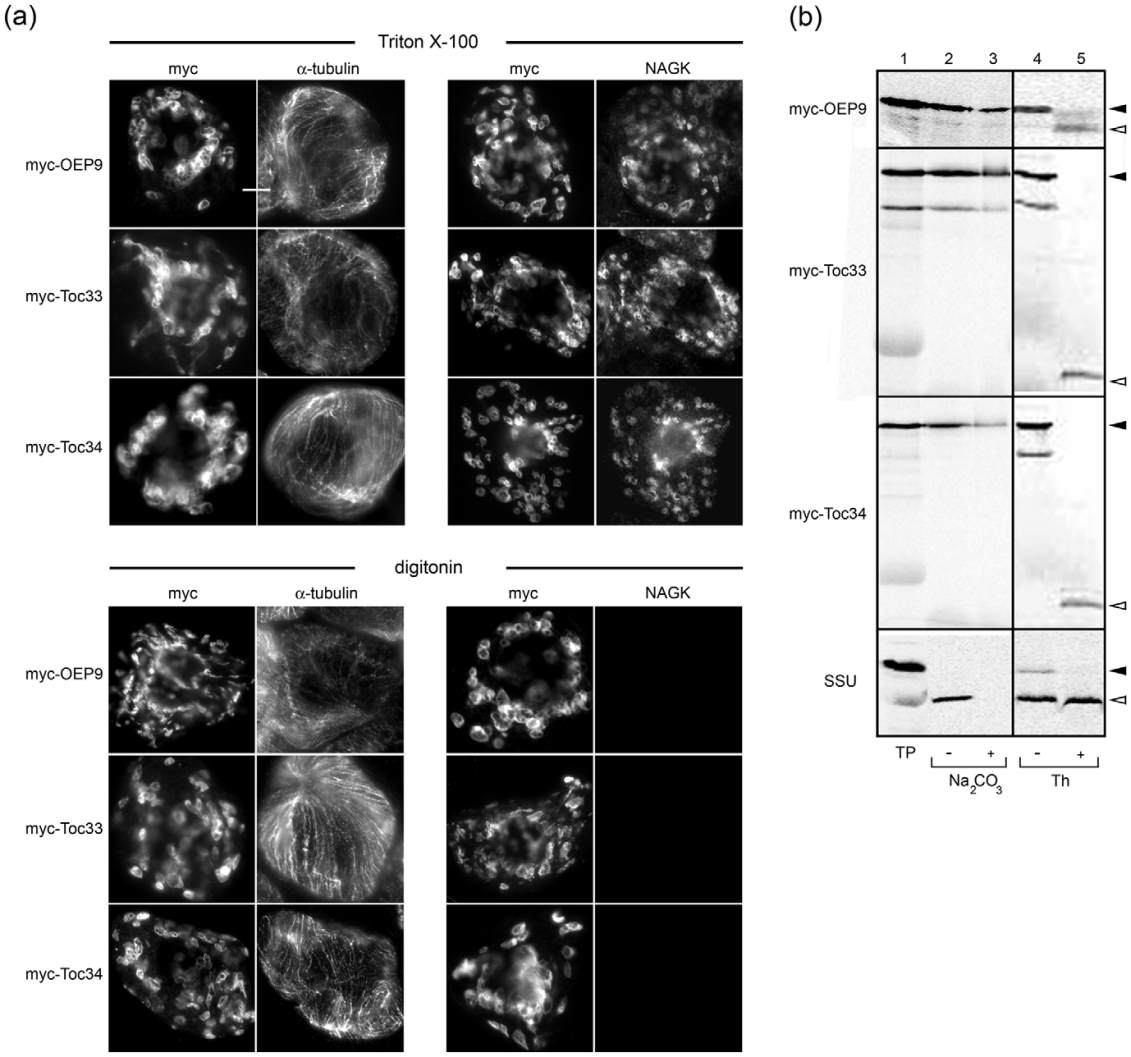
Topology and membrane insertion of OEP9, Toc33 and Toc34. (a) Epi-(immuno)fluorescence micrographs of BY-2 cells transformed with either myc-tagged OEP9, Toc33 or Toc34, differentially permeabilized with either Triton X-100 (top set of panels) or digitonin (bottom set of panels), and then incubated with antibodies raised against (as indicated by the labeling at the top of each column of panels) either the myc epitope, α-tubulin or NAGK. Bar = 10 µm. (b) Insertion of myc-tagged OEP9, Toc33 and Toc34 into chloroplasts *in vitro*. Isolated *Arabidopsis* chloroplasts were incubated with *in vitro* synthesized translation products (TP) including either myc-OEP9, myc-Toc33, myc-Toc34 or SSU then resuspended with Na_2_CO_3_ or incubated with thermolysin (Th). Addition of Na_2_CO_3_ or Th to the reaction mixtures is indicated as (+), omission as (−). Equivalent amounts of each Na_2_CO_3_- or mock-extracted or Th-treated chloroplast membrane sample were subjected to SDS-PAGE/phosphoimaging. The migration in the gel of full-length myc-OEP9, myc-Toc33, myc-Toc34 and the precursor form of SSU are indicated by solid arrowheads, whereas the resulting Th-protected fragments for these proteins, including the mature, processed form of SSU, are indicated with open arrowheads.

To determine whether OEP9, Toc33 and Toc34 were actually oriented in the plastid outer envelope in BY-2 cells in a TA (N_out_-C_in_) manner, cells individually expressing these three N-terminal myc-tagged proteins were differentially permeabilized with either Triton X-100 or digitonin, and then examined by epi(immuno)fluorescence microscopy. As shown in Figure 3a, myc-tagged OEP9, Toc33 and Toc34 were all immunodetected in cells incubated either with Triton X-100, which permeabilizes all cellular membranes, or with digitonin, which permeablizes only the plasma membrane [44]. In the corresponding same cells, however, endogenous stromal NAGK was only immunodetected in cells permeabilized with Triton X-100 and not in cells permeabilized with digitonin, whereas cytosolic α-tubulin was detected in both Triton X-100- and digitionin-permeabilized cells, as expected. Similarly, expressed, non-epitope-tagged OEP was only immunodetected (via antibodies raised against a synthetic peptide in the protein's CTS [refer to Figure 1a]) in cells permeabilized with Triton X-100 and not in cells permeabilized with digitonin (Figure S2e). Taken together, these results confirm that, consistent with a TA topology, the myc epitope and thus the N terminus of OEP9 (and Toc33 and Toc34) is orientated towards the cytosol, while the C terminus of OEP9 faces the intermembrane space.

Membrane insertion, in addition to topological orientation of myc-tagged OEP9, Toc33 and Toc34, was assessed also using an *in vitro* import system with isolated *Arabidopsis* chloroplasts [45]. Figure 3b (lanes 2 and 3) shows that *in vitro* synthesized myc-tagged OEP9, Toc33 and, although to a lesser extent, Toc34, integrated stably into chloroplast membranes, as evidenced by their resistance to extraction with alkaline Na_2_CO_3_. The lack of molecular mass shift for these three membrane-integrated proteins (i.e., when compared to the size of their translation products alone [lane 1]) also confirmed that each protein is devoid of a cleavable transit peptide. Membrane-integrated, myc-tagged OEP9, Toc33 and Toc34 were also confirmed to be orientated in the proper TA (N_out_-C_in_) manner, since treatment of isolated chloroplasts containing these proteins with thermolysin yielded (smaller) protected protein fragments of the expected size, i.e., approx. 6-kDa, 4-kDa and 5-kDa fragments representing the predicted molecular mass of the C-terminal TMD and CTS of OEP9, Toc33 and Toc34, respectively (lanes 4 and 5, Figure 3b). Similar results were observed for the membrane insertion and topology of non-epitope-tagged OEP9 (Figure S2f), reinforcing earlier conclusions, based on *in vivo* localization and topology experiments (see above), that the addition of the myc sequence to OEP9 (i.e., myc-OEP9) does not affect its normal targeting and insertion. Likewise, the results presented here for the membrane insertion and topology of myc-Toc33 and myc-Toc34 *in vitro* are consistent with those published previously for non-epitope-tagged Toc33 and Toc34 [25], [27]. That the precursor form of the soluble small subunit of Rubisco (SSU) was efficiently imported into isolated chloroplasts and properly processed into its mature, thermolysin-protected form (Figure 3b), as expected [46], confirmed the import competence of the chloroplasts used in our *in vitro* import assays.

### Characterization of the targeting information in OEP9, Toc33 and Toc34

**Figure 4:**
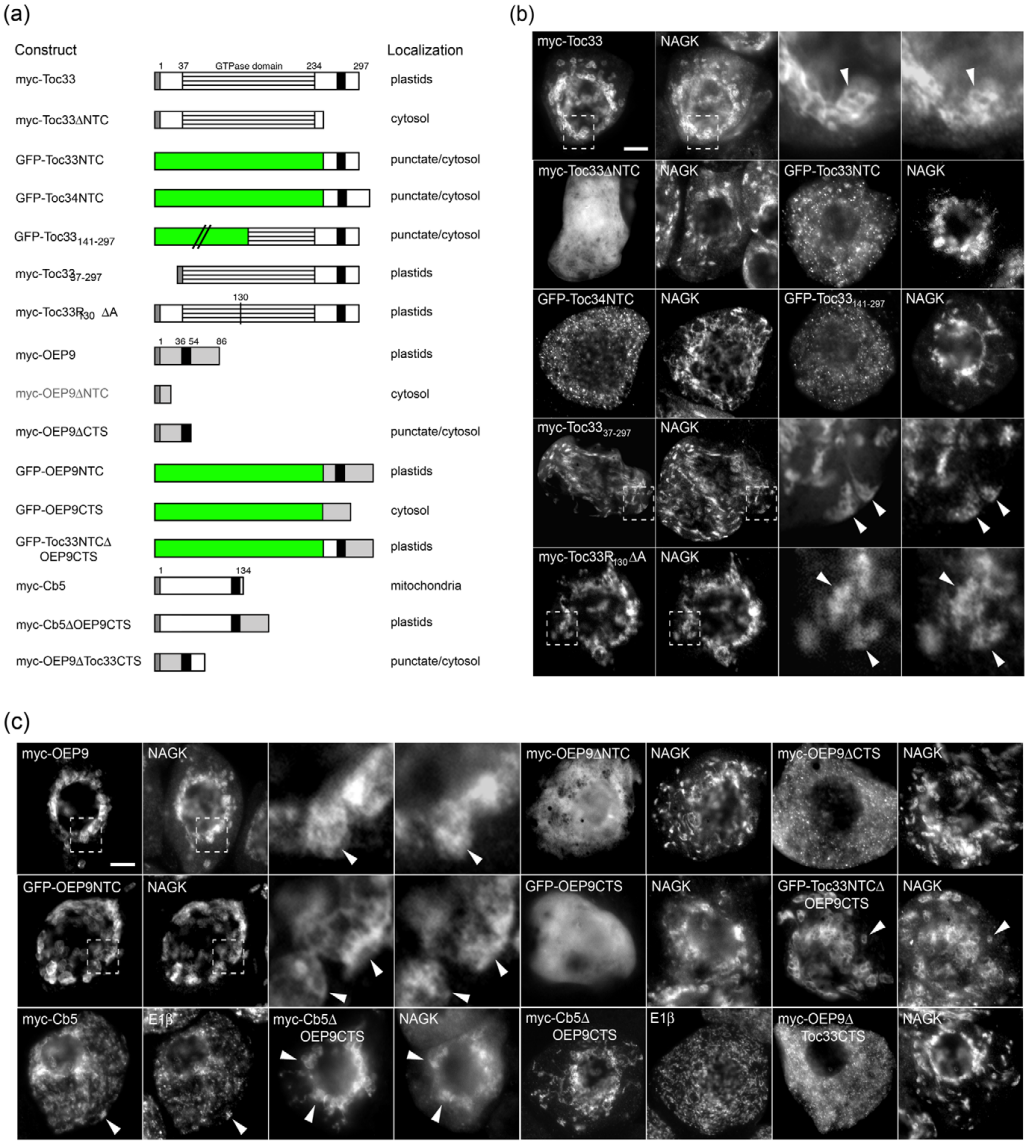
Characterization of the targeting information in OEP9, Toc33 and Toc34. (a) Schematic illustrations of various myc- and/or GFP-tagged wild-type, mutant or chimeric versions of OEP9, Toc33, Toc34 and Cb5, and their corresponding localizations in transformed BY-2 cells. Numbers in the name of some constructs denote the specific amino acid residue(s) that were either fused to the C terminus of GFP or replaced with alanine. Grey and green boxes denote the position of the myc epitope and GFP, respectively. Striped boxes represent the GTPase domains in Toc33 and Toc34, and black boxes represent the single TMDs present in selected proteins. Note that certain constructs included either deletion or fusion (to GFP) of the OEP9, Toc33 or Toc34 CTS or NTC. (b and c) Epi-(immuno)fluorescence micrographs of BY-2 cells transformed (individually) with (b) various Toc33 and Toc34 constructs or (c) various OEP9, Cb5 and Toc33 constructs, all of which are illustrated in (a). Each micrograph is labeled at the top left with the name of either the expressed myc- or GFP-tagged, wild-type or mutant and/or chimeric Toc33, Toc34, OEP9 or Cb5 construct, endogenous plastidial NAGK, or endogenous mitochondrial E1β. Hatched boxes represent the portion of the cells shown at higher magnification in the panels to the right. Arrowheads indicate examples of colocalizations. Bar = 10 µm.

To characterize the specific molecular targeting information required for sorting of OEP9, Toc33 and Toc34 to the plastid outer envelope, we conducted a comprehensive series of *in vivo* targeting experiments (Figure 4) using chimeras consisting of either: i) different portions of each of these three TA proteins fused to GFP serving as a passenger protein; or ii) specific protein domains swapped between OEP9 and either Toc33 or the mitochondrial isoform of Cb5, one of the best-studied plant TA proteins in terms of its targeting and membrane insertion [17]–[19]. We focused mostly on Toc33, rather than Toc34, in these mutational targeting experiments because these two proteins possess a relatively high degree (61%) of amino acid sequence identity [29] and because Toc33 has been less studied in terms of its targeting information, and only using *in vitro*-based assays [27].

As shown in Figure 4b, transiently-expressed myc-Toc33 sorted exclusively to endogenous NAGK-containing plastids, as expected (cf. cells expressing myc-Toc33 and co-stained for NAGK in Figure 2a and 4b). On the other hand, deletion of the so-called ‘NTC domain’ from Toc33, namely the C-terminal region of the protein consisting of the 20 amino acid residues immediately upstream (N terminal) of its TMD, the TMD, and CTS, resulted in the modified protein (myc-Toc33ΔNTC) being mislocalized entirely to the cytosol (Figure 4b). When the NTC of Toc33 was appended to the C terminus of GFP, however, the resulting fusion protein (GFP-Toc33NTC) localized to numerous small punctate structures that are not plastids, as evidenced by the lack of colocalization of GFP-Toc33NTC and NAGK. Instead, as discussed below, these structures containing GFP-Toc33NTC are likely protein aggregates in the cytosol. Similar results were observed when the NTC domain of Toc34 was appended to GFP (GFP-Toc34NTC) (Figure 4b).

That the NTCs of Toc33 and Toc34 were necessary, but not sufficient, for plastid targeting indicated that other important targeting information existed in the N-terminal regions of these proteins. To test this possibility, a chimera consisting of GFP appended to the Toc33 NTC plus an additional ∼100 upstream amino acid residues of Toc33 was constructed (i.e., GFP-Toc33_141–297_). As shown in Figure 4b, GFP-Toc33_141–297_, similar to GFP-Toc33NTC, localized to small punctate structures that were devoid of NAGK. On the other hand, GFP-Toc33_37–297_, consisting of amino acid residues 37 to 297, including the protein's entire GTP-binding (G)-domain fused to GFP, sorted to plastids in a manner similar to full-length myc-Toc33, suggesting that almost the entire Toc33 protein, including its G-domain, is required for proper targeting to plastids. Similarly, a mutant form of myc-Toc33 in which the so-called ‘arginine finger’ residue of its G-domain (i.e., position 130 [47]) was replaced with alanine (myc-Toc33R_130_ΔA) localized exclusively to plastids (Figure 4b). While the results of numerous other studies have shown that this arginine mutation affects the ability of Toc33 to self-dimerize and, thus, function properly as an preprotein import receptor ([48] and references therein), the results obtained here indicate that the plastid targeting of Toc33 itself does not rely on arginine finger-dependent dimerization.

We next characterized the targeting information in OEP9. As shown in Figure 4c, deletion of either the NTC (myc-OEP9ΔNTC) or CTS (myc-OEP9ΔCTS) of OPE9 resulted in mislocalization to the cytosol and to the cytosol and punctate structures, respectively, indicating that the CTS is minimally necessary for proper targeting of OEP9 to plastids. The punctate structures containing myc-OEP9ΔCTS did not co-localize with endogenous marker proteins for mitochondria, peroxisomes or Golgi (Figure S4a), but they did colocalize, at least in some instances, in punctate structures containing the co-expressed fusion protein GFP-OEP7 (consisting of GFP fused to the N terminus to OEP7) (Figure S4b). GFP-OEP7 is known to form protein aggregates in the cytosol of transformed plant cells presumably due to the plastid targeting information near its N terminus being sterically disrupted by the (N-terminal) appended GFP moiety [38], [43]. Thus, partial co-localizations between myc-OEP9ΔCTS and GFP-OEP7 in several punctate structures (Figure S4b), suggests that myc-OEP9ΔCTS (as well as various GFP-Toc33/34 fusion proteins that localize also to punctate structures [see above]) forms protein aggregates in the cytosol due to the disruption of its (C-terminal) plastid targeting information.

Figure 4c shows also that the NTC of OEP9, unlike the NTC of Toc33 or Toc34, possesses sufficient plastid targeting information, since GFP-OEP9NTC (consisting of GFP fused at its C terminus of the OEP9 NTC; Figure 4a) localized exclusively to plastids. The OEP9 CTS alone (residues 54–86), however, was unable to target GFP to plastids, and instead this fusion protein (GFP-OEP9CTS) remained entirely in the cytosol (Figure 4c).

That the CTS of OEP9 is necessary, but on its own not sufficient (see above), for plastid targeting prompted us to test next whether this region contained the protein's key targeting information. Toward this end, we swapped the CTS of Toc33 in the context of GFP-Toc33NTC, which does not sort to plastids (Figure 4b), with the CTS of OEP9, yielding a modified chimeric protein (GFP-Toc33NTCΔOEP9CTS) that localized exclusively to plastids (Figure 4c). The CTS of OEP9 was sufficient also in sorting to plastids a modified version of the mitochondrial isoform of tung tree (*Aleurites fordii*) Cb5 (myc-Cb5ΔOEP9CTS) whereby the three amino-acid-long CTS of Cb5 (-RRK) was replaced with the CTS of OEP9 (Figure 4a). As shown in Figure 4c, while full-length myc-Cb5 sorted to E1β-containing mitochondria and the corresponding myc-Cb5 mutant lacking its CTS (myc-Cb5ΔCTS) mislocalized to the cytosol, as expected [17], myc-Cb5ΔOEP9CTS localized exclusively to plastids. On the other hand, a modified chimeric protein consisting of myc-OEP9 with its CTS replaced with the CTS of Toc33 (myc-OEP9ΔToc33CTS) did not localize to plastids, but instead, similar to myc-OEP9ΔCTS, mislocalized to small punctate structures and the cytosol.

Overall, the data presented in Figure 4 indicate that OEP9, compared to Toc33 and Toc34, contains distinctly different plastid targeting information. The targeting signals in Toc33 and Toc34 being relatively long, consisting of almost the entire protein, including its C-terminal NTC and GTPase domain. By contrast, the targeting signal in OEP9 consisting of only its CTS and the adjacent TMD sequence.

### Detailed characterization of the plastid targeting signal in the CTS of OEP9

**Figure 5:**
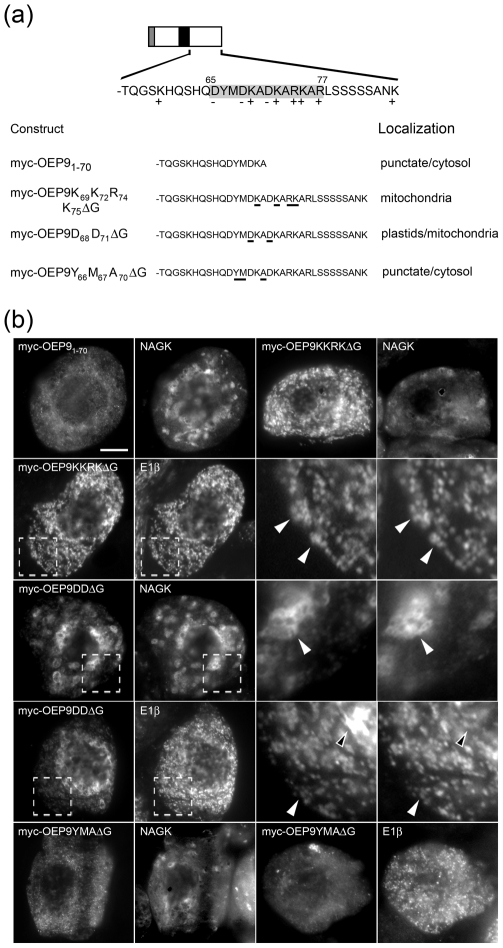
Characterization of the targeting information in the CTS of OEP9. (a) Schematic illustration of myc-tagged OEP9 and the hydrophilic C-terminal sequences (CTSs) of either wild-type or mutant versions of myc-OEP9 and their corresponding localizations in transformed BY-2 cells. Numbers shown above certain amino acids residues in the illustration indicate their relative positions in OEP9 and the cluster of positively (+) and negatively (−) charged residues in the OEP9 CTS are shaded grey. The grey box at the N-terminal end of OEP9 denotes the position of the myc epitope and the OEP9 TMD is colorized black. Numbers in the name of each myc-OEP9 mutant construct denote the specific amino acids in the CTS that were either deleted or replaced with glycine residues. Likewise, the amino acids in the mutant versions of myc-OEP9 that were replaced with glycines are underlined in the corresponding C-terminal sequences. (b) Epi-(immuno)luorescence micrographs BY-2 cells transformed (individually) with various myc-OEP9 constructs, as illustrated in (a). Each micrograph is labeled at the top left with the name of either the expressed wild-type or mutant myc-OEP9 construct, endogenous plastidial NAGK, or endogenous mitochondrial E1β. Note that the numbers in the names of the myc-OEP9 mutant constructs that denote the specific amino acids in the CTS that were replaced with glycine residues (as in [a] and in the Results) were removed in the labels in (b) due to space limitations. Hatched boxes represent the portion of the cells shown at higher magnification in the panels to the right. Solid arrowheads indicate examples of colocalizations; open arrowheads indicate examples of the non-colocalization. Bar = 10 µm.

To gain further insight to the nature of the plastid targeting signal in the CTS of OEP9, we initially deleted the C-terminal half of this region of the protein. As shown in Figure 5, myc-OEP9_1–70_, which lacks the protein's C-terminal 16 amino acid residues (residues 71–86) did not localize to NAGK-containing plastids. Instead, similar to myc-OEP9ΔCTS (Figure 4c), myc-OEP9_1–70_ mislocalized to the cytosol and small punctate structures (presumably protein aggregates), indicating that this deleted portion and/or a combination of the both halves of the CTS are essential for targeting OEP9 to plastids.

An examination of the OEP9 CTS sequence revealed it contains a number of positively- and negatively-charged residues, the majority of which were located between positions 65 to 77 (Figure 5a). Notably, this cluster of charged residues within the CTS was disrupted in the mutant myc-OEP9_1–70_ and is conserved in all the putative homologues of OEP9 (Figure 1a). To assess, therefore, whether these charged residues in the CTS of OEP9 are important for its proper targeting, two mutants were constructed wherein several of either the positively-charged lysine and arginine residues or the negatively-charged aspartate residues were replaced with glycines (Figure 5a). As shown in Figure 5b, the positively-charged mutant myc-OEP9K_69_K_72_R_74_K_75_ΔG mislocalized exclusively to E1β-containing mitochondria and the corresponding negatively-charged mutant, myc-OEP9D_68_D_71_ΔG, also mislocalized partially to mitochondria (i.e., myc-OEP9D_68_D_71_ΔG localized to both plastids and mitochondria). Interestingly, glycine substitutions of other (non-charged) amino acids within the same region of the OEP9 CTS also disrupted the protein's normal targeting to plastids, i.e., myc-OEP9Y_66_M_67_A_70_ΔG mislocalized to punctate structures and the cytosol (Figure 5b). Collectively, these data suggest that the net charge and/or charge distribution of the CTS, as well as the overall three-dimensional configuration of the CTS, mediates the plastid targeting specificity of OEP9.

### AKR2A interacts with OEP9 *in vivo*

Recently, the *Arabidopsis* ankryin repeat-containing protein, AKR2A, was shown to function as an essential cytosolic mediator of OEP biogenesis, acting both as a chaperone to prevent the aggregation of nascent OEPs and as a receptor to facilitate their subsequent targeting from the cytosol to the chloroplast outer envelope [43]. The evidence in support of this dual role for AKR2A provided in part by *in vitro* protein pull-down and/or nuclear mislocalization assays, which demonstrated that AKR2A interacts specifically with various OEPs [43].

**Figure 6:**
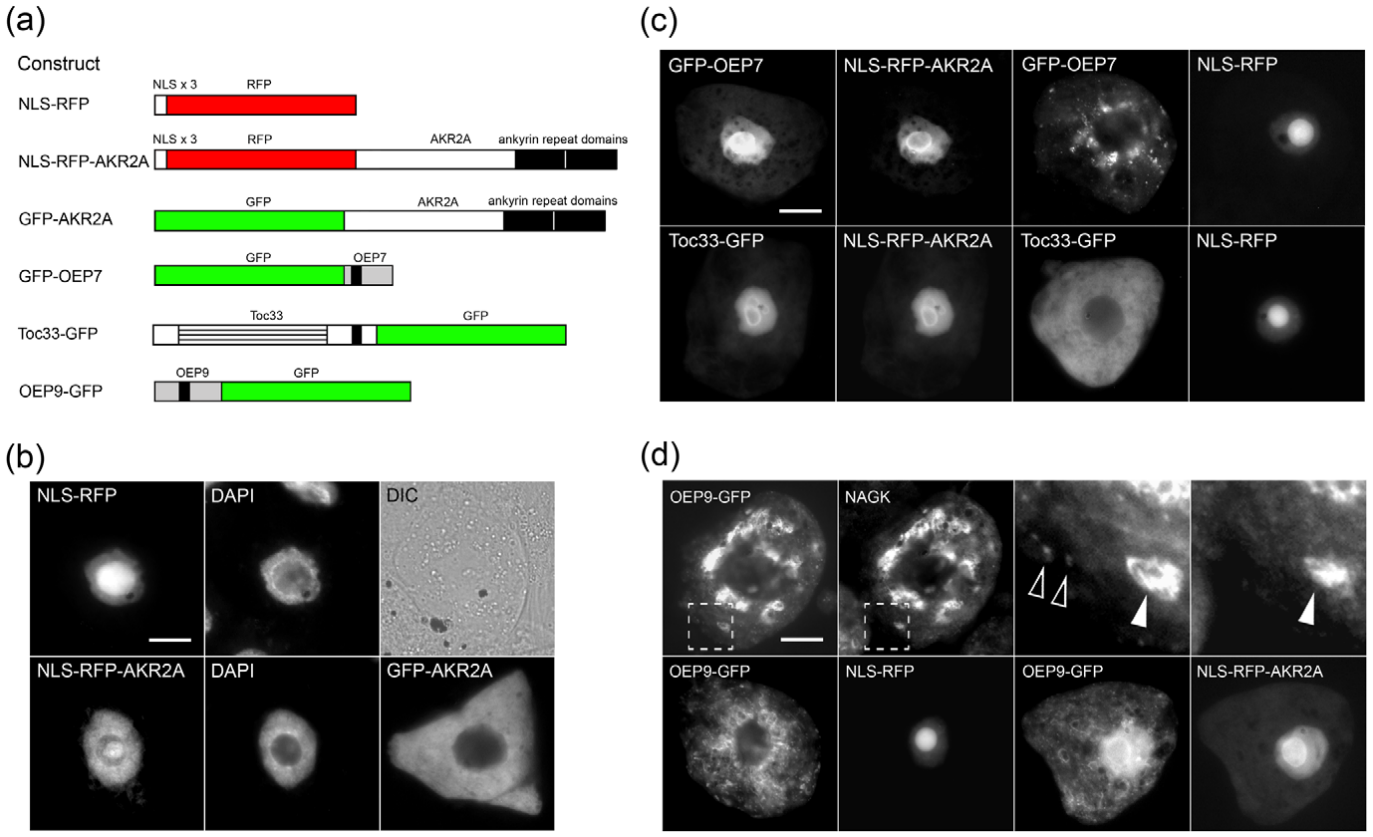
AKR2A mediates the localization of OEP9. (a) Schematic illustrations of NLS-RFP, NLS-RFP-AKR2A, GFP-AKR2A, as well as GFP fusion constructs containing OEP7, Toc33 or OEP9. The three tandem copies of the SV-40 large T antigen NLS (NLS×3) located at the N terminus of NLS-RFP and NLS-RFP-AKR2A are boxed and the dual ankryin repeat domains located at the C terminus of AKR2A are colorized black. Also colorized black is the single TMD present in each of the OEP-GFP (GFP-OEP) fusion proteins. The GTPase domain in the Toc33-GFP fusion protein is represented with a striped box. (b)–(d) Epi-(immuno)fluorescence micrographs of BY-2 cells either (a) transformed with NLS-RFP, NLS-RFP-AKR2A or GFP-AKR2A or co-transformed with (c) GFP-OEP7 or Toc33-GFP, or (d) OEP9-GFP with NLS-RFP or NLS-RFP-AKR2A, all of which are illustrated in (a). Each micrograph is labeled at the top left with the name of either the (co-)expressed fusion protein or, in the corresponding same cells, endogenous plastidial NAGK or DAPI (4′,6-diamidino-2-phenylindole), serving as a stain for the nuclear DNA. Also shown for the NLS-RFP-transformed cell in (b) is the corresponding differential interference contrast (DIC) image. Hatched boxes in (d) represent the portion of the cells shown at higher magnification in the panels to the right; solid arrowheads indicate examples of OEP9-GFP colocalizing with NAGK in plastids; open arrowheads indicate examples of the smaller punctate structures containing OEP9-GFP, but not NAGK. Bar = 10 µm.

To investigate whether AKR2A interacts with OEP9 we also employed a nuclear mislocalization assay. Specifically, we constructed two chimeric proteins (Figure 6a) that consist of three tandem copies of the nuclear localization signal (NLS) from the SV-40 large T antigen [49] fused to either the red fluorescent protein (RFP) alone (NLS-RFP) or to the RFP and AKR2A (NLS-RFP-AKR2A). For comparative purposes, a third chimera was constructed consisting of GFP fused to AKR2A alone (GFP-AKR2A) and that, unlike NLS-RFP-AKR2A, lacks an appended NLS (Figure 6a). Consistent with the intracellular localizations reported previously for these three chimeric proteins in transiently-transformed leaf protoplasts [39], NLS-RFP and NLS-RFP-AKR2A both localized exclusively to the nucleus, while GFP-AKR2A localized to the cytosol in transformed BY-2 cells (Figure 6b), indicating that the NLS was efficient in mislocalizing AKR2A (i.e., NLS-RFP-AKR2A) from the cytosol to the nucleus in these cells.

Also consistent with previously published results [43], NLS-RFP-AKR2A was capable of mislocalizing co-expressed GFP-OEP7 to the nucleus in BY-2 cells (Figure 6c). By contrast, GFP-OEP7 co-expressed with the NLS-RFP, similar to when GFP-OEP7 was expressed on its own, localized to numerous punctate structures that, as discussed above, are likely cytosolic aggregates of this fusion protein [38] (cf. cells expressing GFP-OEP7 and co-expressing GFP-OEP7 and NLS-RFP in Figure S2b and Figure 6c, respectively). Notably, OEP7-GFP sorted to plastids and did not mislocalize to the nucleus when co-expressed with NLS-RFP-AKR2A (Figure S5a), indicating that AKR2A does not bind efficiently to OEP7 when GFP is appended to its N terminus (GFP-OEP7), but does so when GFP is appended to its C terminus (OEP7-GFP); a conclusion that is consistent with previously published data on the functionality, or lack thereof, of the N-terminal plastid targeting signal in OEP7 [38] and why GFP-OEP7 (and not OEP7-GFP) was employed here and elsewhere [43] in nuclear mislocalization assays with AKR2A.

In additional control experiments, Toc33-GFP (consisting of Toc33 fused at its C terminus to GFP; Figure 6a) co-expressed with NLS-RFP-AKR2A localized predominantly to the nucleus (Figure 6c), confirming and extending previous results from *in vitro* pull-down assays showing that Toc33 interacts with AKR2A [43]. On the other hand, when Toc33-GFP was either co-expressed with NLS-RFP or expressed alone it localized to the cytosol and not to plastids (Figure 6c), presumably due to the disruption of the Toc33 plastid targeting information by the C-terminal-appended GFP moiety.

Figure 6d shows that OEP9-GFP, consisting of OEP9 appended at its C terminus to GFP (Figure 6a), localized to both plastids and to numerous punctate structures within the cytosol when expressed on its own. Analogous to the mislocalization of GFP-OEP7, these OEP9-GFP-containing punctate structures likely represent mislocalized aggregates of the fusion protein due to the partial disruption of the OEP9's plastid targeting information by the appended GFP moiety. OEP9-GFP also localized to both plastids and aggregates in the cytosol when co-expressed with NLS-RFP. However, when co-expressed with NLS-RFP-AKR2A, at least a portion of OEP9-GFP (mis)localized to the nucleus, i.e., in addition to being localized to plastids and the cytosolic aggregates, OEP9-GFP also accumulated in the nucleus when co-expressed with NLS-RFP-AKR2A (cf. cells co-expressing OEP9-GFP with NLS-RFP-AKR2A or NLS-RFP in Figure 6d), indicating that OEP9 interacts with AKR2A.

### OEP9, compared to Toc33 and Toc34, requires different membrane-bound proteinaceous factors for integration and displays distinct differences in membrane lipid association

**Figure 7:**
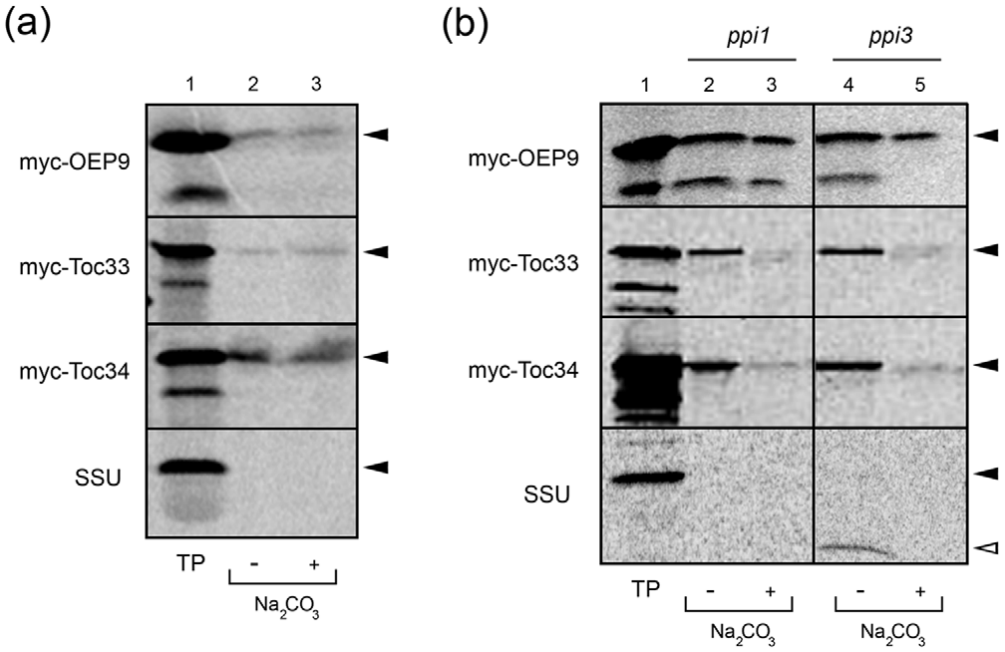
OEP9, Toc33 and Toc34 insert into trypsin-pretreated chloroplasts and *ppi1* and *ppi3* chloroplasts *in vitro*. (a) Insertion of OEP9, Toc33, Toc34 and SSU into trypsin-pretreated chloroplasts *in vitro*. Isolated *Arabidopsis* chloroplasts pre-treated with trypsin were incubated with *in vitro* synthesized translation products (TP) including either myc-OEP9, myc-Toc33, myc-Toc34 or SSU and then resuspended with Na_2_CO_3_ (see ‘Materials and Methods’ for details). Equivalent amounts of Na_2_CO_3_- or mock-extracted chloroplast membranes were then subjected to SDS-PAGE/phosphorimaging. Addition of Na_2_CO_3_ to the reaction mixtures is indicated as (+), omission as (−). The migration in the gel of each protein is marked with an arrowhead to the right of each panel. (b) Insertion of OEP9, Toc33, Toc34 and SSU into *ppi1* and *ppi3* chloroplasts *in vitro*. Chloroplasts isolated from *ppi1* or *ppi3* mutant *Arabidopsis* plants were incubated with *in vitro* synthesized TP including either myc-tagged OEP9, myc-Toc33 myc-Toc34 or SSU. Chloroplasts were then resuspended with or without Na_2_CO_3_ and subjected to SDS-PAGE/phosophorimaging. Addition of Na_2_CO_3_ to the reaction mixtures is indicated as (+), omission as (−). The migration in the gel of each (full-length) protein is marked with a solid arrowhead to the right of each panel, whereas the mature, processed (cleaved) form of SSU, is indicated with open arrowhead. Note that the smaller, additional bands observed in some of the myc-tagged OEP9, Toc33 and Toc34 lanes (e.g., lane 1) were present in varied amounts depending on the translation reaction (cf. lane 1 here and lane 1 in Figure 3) and are, as described previously for Toc33 [27] and psToc34 [24], likely truncated versions of these proteins due to internal translation initiation(s).

Given our results indicating that OEP9, similar to other OEPs, relies on AKR2A as a mediator (i.e., chaperone/receptor) for its targeting from the cytosol to plastids (Figure 6), we examined next whether other protein(s), if any, are responsible for the subsequent insertion of OEP9 into the plastid outer envelope membrane. Toward this end, OEP9, Toc33 and Toc34 were compared initially for their ability to insert into isolated chloroplasts that were treated with the protease trypsin prior to the insertion reaction and, thus, removed surface-exposed outer membrane proteins including candidate receptor(s). As shown in Figure 7a, only a portion of *in vitro* synthesized, radiolabeled myc-tagged OEP9, Toc33 and Toc34 bound to and stably integrated into (as evidenced by their resistance to extraction with Na_2_CO_3_) trypsin-pretreated chloroplasts. That is, compared to the behavior of these three proteins in reactions containing untreated intact chloroplasts (refer to Figure 3b, lanes 2 and 3), their binding and integration into trypsin-pretreated chloroplasts was substantially reduced, although to a much lesser extent for myc-Toc34 (Figure 7a). The import and processing of SSU, however, was completely abolished by the pre-treatment of chloroplasts with trypsin (Figure 7a). These latter results confirm that the protease had efficiently degraded proteins of the Toc complex, since the import of SSU is well known to be Toc complex-dependent [29], [50].

We demonstrated also that the integration, but not the binding, of myc-Toc33 and myc-Toc34 into chloroplast outer envelope membranes was significantly reduced when *in vitro* import reactions with either of these two proteins contained chloroplasts isolated from *ppi1* or *ppi3 Arabidopsis* mutant plants that lacked (via a T-DNA insertion) Toc33 [29] or Toc34 [51], respectively (Figure 7b). These data indicate that Toc33 and Toc34 themselves serve as receptor proteins involved in their proper insertion. OEP9, however, does not appear to depend on the Toc33 or Toc34 receptors, since it bound and integrated (Figure 7b), as well as orientated (based on thermolysin protection assays) in the proper (TA) manner (Figure S6), into both *ppi1* and *ppi3* chloroplasts in a manner similar to that for wild-type chloroplasts (cf. Figure 3b, 7b and S6). Instead, data presented in Figure 7a indicate that the OEP9 is dependent, at least in part, on some other surface-exposed proteinaceous factor(s). On the other hand, SSU was properly imported into *ppi3* chloroplasts, but was not into *ppi1* chloroplasts (Figure 7b), consistent again with previous studies indicating that this protein, like other photosynthetic proteins, relies more so on Toc33 (than Toc34) for its import [29], [50], [51].

The observation that a portion of OEP9, Toc33 and, to a greater extent, Toc34, inserted into protease-pretreated chloroplasts (Figure 7a) might be due to direct protein-lipid interactions and, thus, we tested whether these three TA proteins can bind to synthetic membrane lipids *in vitro*. Specifically, translation reactions containing myc-tagged OEP9, Toc33 or Toc34 were incubated with or without protein-free lipid membranes (liposomes) containing an average lipid composition similar to that of the chloroplast outer envelope membrane [52]. All of the samples were subsequently subjected to sucrose gradient centrifugation followed by fractionation of the gradient into those containing either the liposomes and liposome-bound proteins (fractions 1 and 2), unbound proteins that remained in a specific portion of the sucrose gradient (fraction 3), the load fraction (fraction 4) or aggregated proteins that pelleted to the bottom of the gradient (fraction 5) [53].

**Figure 8:**
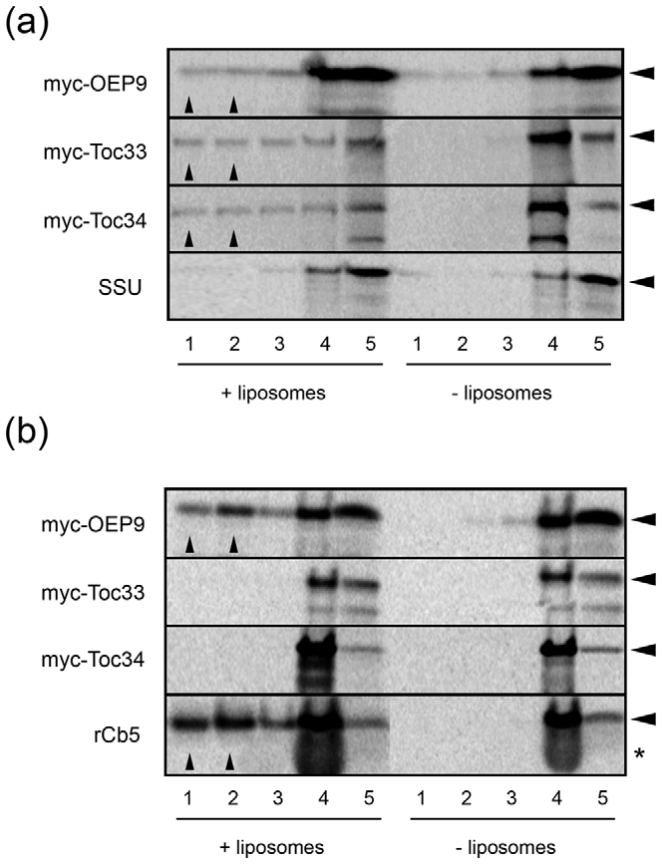
Insertion of OEP9, Toc33 and Toc34 into protein-free liposomes with varied lipid compositions *in vitro*. *In vitro* synthesized myc-OEP9, myc-Toc33, myc-Toc34 or SSU were incubated with (+) our without (−) liposomes that contained a lipid composition similar to that of either (a) the chloroplast outer envelope membrane or (b) mitochondria; see ‘Materials and Methods’ for the lipid composition of liposomes. Following incubation, samples were subjected to sucrose-gradient centrifugation and the resulting gradients were fractionated (as described in the ‘Results’), and then equivalent amounts of all fractions (1–5) were subjected to SDS-PAGE/phosophorimaging. Note that the migration in the gel of each protein examined is indicated by the arrowheads at the right side of the panels in (a) and (b). The asterisk at the right side of the panel in (b) indicates the migration position of rabbit globin in the load fraction (fraction 4) of samples containing rCb5.

As shown in Figure 8a, a portion of the myc-tagged OEP9, Toc33 and Toc34 added to the incubations was recovered in gradient fractions containing chloroplast-like liposomes (fractions 1 and 2, solid arrowheads), indicating that all three proteins were binding directly to this lipid bilayer. However, their binding efficiency to chloroplast-like liposomes varied considerably, i.e., while a substantial portion of Toc33 and Toc34 proteins were recovered in fractions with liposomes (fractions 1 and 2) compared to the gradient and load fractions (fractions 3 and 4), the majority of soluble OEP9 remained in the load and bottom fractions (fractions 4 and 5). Moreover, a portion of OEP9 was recovered in the top soluble fractions (fractions 1 and 2) of gradients without liposomes. Overall, these data indicate Toc33 and Toc34 bind much more efficiently to the chloroplast-like liposomes than OEP9. Shown also in Figure 8a, the majority of the soluble control protein SSU remained in the load and bottom fractions in gradients with or without liposomes, indicating that SSU, consistent with previous results [54], does not interact with chloroplast-like liposomes.

We next assessed whether the targeting to chloroplast-like liposomes of Toc33, Toc34, and, although to a much lesser extent, OEP9 was specific for this lipid bilayer, since previous studies with psToc34 revealed its insertion into liposomes was dependent on the presence of lipids unique to the plastid outer envelope, namely the non-bilayer lipids mono/digalactosyldiacylglycerides (MGDG/DGDG), and on the concentration of anionic lipids, such as phosphatidylglycerol (PG) [24]. We therefore tested the ability of OEP9, Toc33 and Toc34 to bind liposomes that, unlike chloroplast-like liposomes, were devoid of MGDG, DGDG and PG, and possessed different amounts of other lipids that, overall, yielded a composition similar to mitochondrial membranes [53]. Sucrose gradient flotation assays were employed for these experiments as described above, but, rather than SSU, an ER isoform of rat Cb5 (rCb5) served as a control protein, since this TA protein targets *in vitro* to any membrane, including synthetic liposomes [13], [53]. As shown in Figure 8b, neither myc-Toc33 nor myc-Toc34 bound to the mitochondrial-like liposomes, consistent with previous results for psToc34 [24]. By contrast, myc-OEP9 bound to the mitochondrial-like liposomes and did so in a manner similar to rCb5 (Figure 8b). Taken together, the data presented in Figure 8 suggest that OPE9, compared to Toc33 and Toc34, displays differences in its preference for binding membrane lipids, and that this behavior may serve as an important determinant in the targeting specificity of these three TA proteins.

## Discussion

### The sorting of TA proteins to plastids involves at least two distinct pathways

Plastids participate in a wide array of essential metabolic processes, all which rely on the acquisition of distinct nuclear-encoded protein components from the cytosol, and as such, the protein composition of the organelle is influenced by both nuclear gene expression and the activity of intracellular targeting pathways specific for plastid biogenesis. In fact it is now well appreciated that multiple import pathways serve in the uptake of both soluble and membrane-bound proteins into plastids [28]. However, compared to other proteins our understanding of plastid TA protein biogenesis is lacking. Given the importance of TA proteins in other critical aspects of cell metabolism and physiology, we undertook a comparative analysis of the targeting and insertion mechanisms of three plastid TA proteins, including Toc33 and Toc34, both of which function as Toc complex-receptor GTPases, and OEP9, a newly-identified TA protein of unknown function. Overall, our results provide evidence in support of at least two pathways for plastid TA biogenesis that are distinguished by the nature of their molecular targeting signals and the membrane protein and lipid components involved. These findings should now not only facilitate a more detailed analysis of these membrane components, some of which may be shared in terms of their underlying biochemical mechanisms, but also complement the growing body of evidence for the complex diversity of plastid protein sorting pathways, as well as the diversity of sorting pathways for TA proteins localized to other organelles (e.g., mitochondria [7] and ER [55]).

### OEP9 is integrated in the plastid outer envelope in a TA manner and may be involved in ribosome biosynthesis in roots

OEP9 was one of over 500 *Arabidopsis* candidate TA proteins identified in a recent bioinformatics screen [32] based primarily on the three main structural characteristics that have traditionally defined the TA protein family, including: 1) the presence of a single putative TMD within the C-terminal ∼50 amino acid residues; 2) the absence of any other TMDs; and 3) the lack of an N-terminal hydrophobic secretory signal sequence [1]. Consistent with each of these characteristics, we showed here using differential detergent permeabilization and protease protection assays (Figure 3) that OEP9 is stably integrated in the chloroplast outer envelope in a TA manner (i.e., N_out_-C_in_). This interpretation of OEP9's TA topology was reinforced by results from a parallel series of assays with Toc33 and Toc34 (Figure 3), all of which were in full agreement with the previously reported TA topology of these two proteins [25], [27].

Similar to many other OEPs [35], [56], the function of *Arabidopsis* OEP9 is unknown. Nevertheless, the existence of conserved OEP9 homologues in other diverse plant species (Figure 1c) and the absence of homologues in non-plant organisms, suggests that its function(s) is plant specific. Indeed, some indirect evidence obtained from various web-based *Arabidopsis* (co)expression datasets supports the possibility that OEP9 functions in the plastids of root cells and in plastid ribosome biosynthesis (Figure S1). Whether OEP9 is actually involved in plastid ribosome biogenesis in roots, however, remains to be tested experimentally; a task that will likely require inducible RNAi mutants of OEP9, since knock-out (T-DNA) mutants of this gene or its paralogue (At1g80890) are not available, suggesting also that OEP9 is essential for plant growth and development.

### Properties of the OEP9 targeting signal

Since plant cells possess an additional organelle (the plastid) that is absent in most other eukaryotic cells (e.g., yeast and mammals), there is an added level of complexity in the intracellular trafficking system for plant TA proteins that warrants a close examination of the targeting signals involved. For almost all TA proteins, regardless of their organelle destination, the initial targeting event is mediated by *cis*-acting sequences within the C-terminal region of the protein [2]. Consistent with this paradigm, the OEP9 CTS and TMD together are both necessary and sufficient for targeting the protein from the cytosol to plastids (Figure 4). However, these sequences within OEP9 also appear to play distinct roles: the CTS contains the protein's key plastid targeting information and the TMD, which in addition to being required for thermodynamic association and integration into membranes, possesses general physico-chemical properties, such as overall hydrophobicity, length, and/or propensity to form an α helical structure, that act together to convey the proper context for the CTS to function as a targeting signal. Perhaps the best support of this conclusion is that the CTS of OEP9 on its own is not sufficient for targeting GFP to plastids, but is sufficient in re-targeting the mitochondrial isoform of Cb5 or the NTC domain of Toc33 fused to GFP to plastids (Figure 4c). By contrast, the Toc33 NTC domain on its own was not sufficient for targeting GFP to plastids (Figure 4b). As discussed below, these latter data indicate that Toc33 (and Toc34) possesses a different type of targeting signal than OEP9 since it relies on additional targeting information present within the N-terminal GTPase domain of the proteins.

Inspection of the CTS sequences of OEP9 and putative OEP9 homologues in other plant species revealed several conserved features that possibly represent distinct targeting signal motifs. For instance, all of these proteins possess a cluster of conserved positively- and negatively-charged amino acid residues (residues 65–77; Figure 5a) that are similar to the charged residues known to be important for the proper sorting of other OEPs, namely *Arabidopsis* OEP7 and OEP64 [38], [42]. Interestingly, both OEP7 and OEP64 possess a single TMD, but unlike OEP9, it is located near the protein's N terminus and yields an N_intermembrane space_-C_cytosol_ orientation in the outer envelope membrane [38], [42]. Moreover, the clusters of charged residues in OEP7 and OEP64 have been implicated in preventing interaction with SRP and thus entry into the Sec61 co-translational pathway of the secretory system. We also found that the charged residues in OEP9 (CTS) are critical for its proper targeting to plastids (Figure 5), suggesting that OEP9 shares the same targeting information and, hence, as discussed below, utilizes the same plastid biogenetic pathway as OEP7/64. One important difference between OEP9 and OEP7/64, however, is that mutations to certain charged residues in the CTS of OEP9 resulted in the modified proteins being mistargeted to mitochondria, rather than to the secretory system (Figure 5). This difference in (mis)targeting is most likely due to both OEP7 and OEP64 possessing an N-terminal TMD that, when their charged residues are mutated, engages the SRP/Sec61 co-translational pathway, whereas OEP9 (wild-type or mutant) possesses a C-terminal TMD that emerges from the ribosome only after the termination of translation and, thus, targets strictly in a SRP-independent post-translational manner.

The mitochondrial mislocalization of OEP9 mutants with alterations to certain charged residues within the CTS (Figure 5) also suggests that the TA targeting pathways for chloroplasts and mitochondria are independent, but competing, and that the specific sorting of OEP9, as well as other TA proteins, to either of these two organelles (or to other organelles) is not based strictly on the overall net positive charge of the CTS. While the actual distribution of the charges in the CTSs of these proteins may be an important aspect in mediating targeting specificity, the basic mechanism(s) that underlies the proper sorting of TA proteins in plant cells does not appear to match that in mammals, wherein a net positive charge in the CTS conveys sorting to mitochondria and a net negative or null charge conveys sorting to the ER [2]. It seems instead that plant TA protein targeting signals have acquired additional information that ensures higher fidelity association with the correct organelle [17], [18]. Consistent with this premise, mutational analyses of the OEP9 CTS revealed that, in addition to the charged-related characteristics, the overall secondary and/or three-dimensional configuration of this region appears to play an important role in plastid targeting specificity (Figure 5). This is potentially an important featureof the OEP9 CTS, since at least some protein structure prediction programs indicated that this region, as well as the N terminus of the protein, has the propensity to be intrinsically disordered (Figure 1a) and disordered segments in other proteins can serve as specific binding/recognition elements and/or flexible linkers involved in macromolecular assembly [57]. Spectroscopic and prediction-based structural modeling of the CTS of wild-type and mutant OEP9 proteins, as well as large-scale and systematic mutational analyses of the putative targeting signals in the CTSs of other (predicted) plastid TA proteins [32] are now being planned in order to determine whether the putative unstructured domains and/or physico-chemical and sequence-specific features in OEP9 are functionally conserved.

### Role of the GTPase domain in the targeting and membrane insertion of Toc33 and Toc34

Compared to OEP9 and most other TA proteins examined to date, Toc33 and Toc34 appear to be unique in that targeting is not mediated only by sequences within their C-terminal TA sequence. That is, while the NTC sequences of Toc33 or Toc34 are necessary for their targeting to plastids, they are not sufficient in redirecting GFP from the cytosol to plastids (Figure 4b). Only when the entire G-domain of Toc33, along with the NTC region, was fused to GFP was targeting to plastids observed (Figure 4). Taken together, these data are consistent with previous *in vitro* studies indicating that the G-domain of Toc34, along with the TMD and CTS sequences, is important to varying degrees for insertion into isolated chloroplasts [22], [25], [26]. Interestingly, we observed also that a G-domain-mutant version of Toc33 (myc-Toc33R_130_ΔA), which exists primarily as a monomer *in vitro* [47], [48], targets to plastids *in vivo* in a manner similar to its wild-type counterpart (Figure 4b). Thus, while it appears that the G-domains of Toc33 and Toc34 are, at a minimum, critical structural determinants important for maintaining the overall targeting- and/or insertion-competent conformation of these receptor proteins, the so-called ‘arginine fingers’ within these G-domains and, hence, the self-dimerization process that they presumably mediate [48], is not a prerequisite for proper targeting. However, since Toc33/34 at the chloroplast surface are important for mediating their own insertion (Figure 7b; see below), resident Toc33/34 homologs in BY-2 cells, which presumably contain a corresponding intact arginine finger, may account, in part, for the successful plastid targeting of myc-Toc33R_130_ΔA. It is also possible that the apparent differences in the role(s) of the ‘arginine fingers’ in targeting and/or insertion/assembly versus homodimerization of Toc33/34 reflects the complex nature of (TA) OEP membrane biogenesis in general and the different approaches (*in vivo* versus *in vitro*) employed to study this multi-step process.

### Role for ‘kinetic factors’ in the organelle-specific targeting and membrane insertion of OEP9, Toc33 and Toc34

In recent years, considerable progress has been made towards understanding the biogenetic pathways responsible for the intracellular localizations of TA proteins [2]. Based almost entirely on studies carried out with yeast and mammalian model systems, and with TA proteins that localize to mitochondria, peroxisomes or ER, the current working model for TA protein biogenesis involves two main steps: (i) delivery of the nascent protein from its sites of syntheses in the cytosol to the surface of the appropriate organelle, a process that must also ensure the avoidance of interaction with inappropriate organelles; and (ii) the subsequent insertion of the TA protein into its proper membrane bilayer. Both of these steps rely on, depending on the TA protein, one or more so-called ‘kinetic factors’ (e.g., *cis*-acting targeting and insertion sequences, cytosolic proteins, membrane proteins and/or lipids, etc.) that ultimately serve to accelerate the integration and, thus, the retention of the TA protein into its proper organelle membrane destination [2].

In the case of plastid TA proteins, our results and those presented elsewhere [39] indicate that the first step in their biogenetic pathway is mediated, at least in part, by the cytosolic chaperone/receptor AKR2A. That is, our data from nuclear mislocalization assays suggests that AKR2A controls the intracellular distribution *in vivo* of both OEP9 and Toc33, as well as the non-TA (control) protein OEP7 (Figure 6). While this conclusion for AKR2A and OEP9 likely requires addition experimental support, it is reasonable to presume that ARK2A does not appear to function as a general mediator of other (non-plastid) membrane proteins, including TA proteins, since AKR2A did not interact *in vivo* with the mitochondrial isoform of Cb5 (Figure S5b) or *in vitro* with the 22 kDa peroxisomal membrane protein or mitochondrial TOM20 [43]. On the other hand, AKR2A interaction specifically with OEPs appears to be mediated by the plastid targeting sequences since AKR2A does not bind *in vitro* to OEP7 or OEP64 that are devoid of their targeting signals [43] or *in vivo* either to OEP7 when the protein's targeting signal is sterically blocked by an N-terminal appended GFP moiety [38] (Figure S5a) or to OEP9 lacking its CTS (Figure S5c). The mechanism by which AKR2A recognizes TA and non-TA OEPs and how AKR2A functions as a chaperone to maintain nascent OEPs in a targeting- and insertion-competent state are open questions.

We showed also that membrane-bound protein factors play an important role in the insertion of OEP9, Toc33 and Toc34 into the plastid outer envelope. However, the specific membrane proteins involved and, thus, the underlying mechanisms that mediate the insertion of these three TA proteins appears to be different for OEP9 compared to that for Toc33 and Toc34. For instance, while binding to and insertion into the membrane was sensitive to trypsin pretreatment of chloroplasts for all three TA proteins *in vitro* (Figure 7a), only Toc33 and Toc34 did not insert into chloroplasts isolated from mutant plants lacking Toc33 (*ppi1*) or Toc34 (*ppi3*), albeit less so for Toc34 (Figure 7b). These data suggest that Toc33 and Toc34 themselves are essential for their insertion. Moreover, that Toc33 and Toc34 still bound, but did not integrate into, *ppi1* or *ppi3* chloroplasts (Figure 7b) and that the targeting efficiency of Toc34 to trypsin-treated chloroplasts *in vitro* was greater than that of Toc33 (Figure 7a) is consistent with previous conclusions that the biogenesis of these two receptor proteins relies on additional, perhaps different, membrane proteins [26], [30].

While the identity of the membrane protein factor(s) involved in the binding and/or insertion of OEP9 into the plastid outer envelope also remain to be determined, both Toc33 and Toc34 are not likely candidates in this regard since OEP9 inserts efficiently and in the correct (TA) topology into *ppi1* and *ppi3* chloroplasts (Figure 7b and S6), supporting further the notion that the mechanism of insertion of OEP9 is different than that of Toc33 and/or Toc34. Indeed, since OEP9 appears to share the same targeting information as OEP7 (see above), it may utilize the same insertion machinery, i.e., Toc75, the protein-conducting channel of the Toc complex that serves, in addition to its role in Toc complex-mediated preprotein translocation, in the membrane insertion of the OEP7 homolog from pea (OEP14) [58]. By contrast, Toc75 does not appear to participate in the insertion of psToc34 into the chloroplast outer envelope [24].

In addition, OEP9, Toc33 and Toc34 appear to rely on membrane lipids, but yet they do so in different ways. For instance, while all three TA proteins bound to protein-free liposomes with a composition that resembled that of the chloroplast outer envelope, OEP9 did so much less efficiently (Figure 8a). By contrast, OEP9, but not Toc33 and Toc34, bound to mitochondrial-like liposomes (Figure 8b). These results, combined with those published previously for the specific insertion of psToc34 into protein-free chloroplast-like liposomes, but not into isolated mitochondria [24], and the proposed role of lipids in the targeting specificity of TA proteins in general [2], suggests that the unique lipid composition of the chloroplast outer envelope allows Toc33 and Toc34 to discriminate between the surface of chloroplasts and that of other incorrect organelles. An interaction that may also help explain, in part, the evolution of a targeting process for these two (receptor) proteins that is dependent on themselves.

In contrast to Toc33 and Toc34, membrane lipids of the chloroplast outer envelope membrane appear to serve primarily to mediate normal thermodynamic association and integration of OEP9, and, therefore, protein factors (e.g., AKR2A and possibly Toc75) likely determine its plastid-specific targeting and integration. This premise is similar to the model developed for the ER-specific isoform of rCb5, which inserts into all membranes in a cell free system, but targets exclusively to the ER *in vivo*, presumably by the action of (cytosolic) protein factors that prevent its nonspecific insertion into other (incorrect) organelle membranes [12]. While this proposed thermodynamic role for membrane lipids in OEP9 biogenesis remains to be confirmed experimentally, it is tantalizing to speculate that the targeting of OEP9 to mitochondrial-like liposomes compared to chloroplast-like liposomes *in vitro* (Figure 8) also reflects an underlying affinity of this protein for certain membrane lipids that may be present at specific sites or domains in the chloroplast outer envelope. For instance, if one considers that the lateral distribution of lipids in the chloroplast outer envelope is likely not uniform [48], [58], it is possible that specific lipid domains exist within this membrane and that these, in combination with certain protein factors, help mediate the proper association and integration of OEP9 into the plastid outer envelope membrane.

## Materials and Methods

### Recombinant DNA procedures and reagents

Standard recombinant DNA procedures were preformed as described by Sambrook et al [59]. Molecular biology reagents were purchased from New England Biolabs Ltd. (Pickering, Canada) and Invitrogen Canada Inc. (Burlington, Canada) and plasmid DNA was isolated using commercially available kits either from Qiagen (Mississauga, Canada), Invitrogen, or Bio-Basic Inc. (Markham, Canada), all in accordance with the manufacturer's instructions. All DNA constructs were verified using dye terminated cycle sequencing preformed at either Arizona State University DNA Laboratory (Tempe, AZ) or the University of Guelph Genomics Facility (Guelph, Canada). Plasmid DNA mutagenesis reactions were carried using the QuikChange site-directed mutagenesis kit (Stratagene, La Jolla, CA). Synthetic oligonucleotides were synthesized by either Sigma-Genosys Canada (Oakville, Canada) or University of Guelph Laboratory Services (Guelph, Canada).

### Construction of Plasmids

A complete description of all plasmids used in this study and a list of the sequences of oligonucleotide primers used in plasmid constructions are provided in Materials and Methods S1 and Table S1, respectively.

### Tobacco BY-2 cell cultures and microprojectile bombardment

Tobacco (*Nicotiana tabacum* cv BY-2) and *Arabidopsis thaliana* (var Lansberg erecta) suspension cell cultures were maintained and prepared for biolistic bombardment as described previously [60]. Transient transformations, including those involving *Arabidopsis* leaf epidermal cells (Figure S2c), were performed using 10 µg of plasmid DNA (or, with one exception [see below], 5 µg of each plasmid for co-transformations) with a biolistic particle delivery system (Bio-Rad Laboratories Ltd., Mississauga, Canada) [44]. For nuclear mislocalization assays, 500 ng of plasmid DNA encoding the GFP fusion protein(s) was used for co-transformations. Following bombardment, cells were incubated for 6–20 h to allow for expression and sorting of the introduced gene product(s) and then processed for immunofluorescence microscopy.

### Immunofluorescence microscopy

Biolistically bombarded tobacco Bright Yellow-2 (BY-2) or *Arabidopsis thaliana* (var Landsberg erecta) suspension-cultured cells were processed for immunofluorescence microscopy as described by Lingard et al [60]. Briefly, both cells were fixed in 4% (w/v) formaldehyde, and then incubated for 2 h with either (for BY-2 cells) 0.01% (w/v) pectolyase Y-23 (Kyowa Chemical Products, Osaka, Japan) or (for Arabidopsis cells) 0.03% (w/v) cellulysin (Calbiochem) and 0.1% (w/v) pectinase (Sigma-Aldrich Ltd., Oakville, Canada). Thereafter, cells were permeabilized with either 0.3% (v/v) Triton X-100 or 25 ug/mL digitonin (Sigma-Aldrich Ltd.) for 30 min. Primary antibodies and sources were as follows: custom rabbit anti-OEP9 antibodies were raised against a keyhole limpet hemocyanin-conugated synthetic peptide corresponding to the OEP9 amino acid sequence DKADKARKARLSSSSSANK (residues 68 to 86 [refer to Figure 1a]) (Cedarlane Laboratories Ltd., Hornby, Canada); mouse anti-myc antibodies in hybridoma medium (clone 9E10; Princeton University Monoclonal Antibody Facility, Princeton, NJ); rabbit anti-*Arabidopsis* N-acetyl glutamate kinase (NAGK) [22]; rabbit anti-pea E1β [41]; rabbit anti-cottonseed catalase [61]; rabbit anti-pea reversibly glycosylated polypeptide [62]; and mouse anti-α-tubulin (Sigma-Aldrich Ltd). Fluorescent dye-conjugated secondary antibodies sources were as follows: goat anti-mouse and goat anti-rabbit Alexa 488 and goat anti-rabbit Cy5 (Invitrogen); goat anti-mouse and goat anti-rabbit rhodamine red-x (Jackson ImmunoResearch Laboratories, Inc., West Grove, PA).

Epifluorescent images of suspension cells were acquired using a Zeiss Axioscope 2 MOT epifluorescence microscope (Carl Zeiss Inc., Thornwood, USA) with a Zeiss 63X Plan Apochromat oil-immersion objective. Image capture was performed using a Retiga 1300 charge coupled device camera (Qimaging, Surrey, Canada) and Openlab 5.0 software (Improvision, Waltham, MA). CLSM images were acquired using a Leica DM RBE (Leica Microsystems Inc., Richmond Hill, Canada) microscope with a Leica 63x Plan Apochromat oil-immersion objective a Leica TCS SP2. Fluorophore emissions were collected sequentially in double-labelling experiments; single-labelling experiments exhibited no detectable crossover at the settings used for data collections. Confocal images were acquired as single optical sections and saved as 512×512 pixel digital images. Note also that epifluorescence images All fluorescence images of cells shown in the figures are representative of >50 independent (transient) transformations from at least two independent transformation experiments. Figure compositions were generated using Northern Eclipse (v. 5.0) software (Empix Imaging Inc., Mississauga, Canada) and Adobe Photoshop CS (Adobe Systems Canada, Etobicoke, Canada).

### Arabidopsis growth conditions

All wild-type and mutant (*ppi1* and *ppi3*) *Arabidopsis* plants were of Columbia-0 ecotype. *ppi1* and *ppi3* seeds were provided by J. Froehlich (Michigan State University). Seeds were surface-sterilized and sown on Petri plates containing 4.3 g/L Murashige and Skoog salt and vitamin mix with buffer (Bioshop Canada Inc., Burlington, Canada), 10 g/L sucrose and 0.8% (w/v) agar as previously described [45]. Seeds were then chilled at 4°C and grown under a long-day cycle (16 h light, 8 h dark) at 20–25°C until being harvested ∼14 days after germination for chloroplast isolations (see below).

### Targeting to chloroplasts and liposomes *in vitro*

*Arabidopsis* chloroplasts were isolated as described by Wang et al [63]. Phospholipid vesicles (liposomes) with various lipid content were prepared by extrusion in 10 mM Tris-HCl (pH 7.5) as described previously [53]. Phosopholipid vesicles of chloroplast-like composition (based on the outer envelope of chloroplasts from spinach [52] with the exception that 6% sulfoquinovosyl diacylglyerol was omitted) contained (as moles percent) 30∶20∶32∶10∶6∶2 digalactosyldiacylglyceride (DGDG)/monogalactosyldiacylglyceride (MGDG)/phosphatidylchloine (PC)/phosphatidylglycerol (PG)/phosphatidylinositol (PI)/phosphatidylethanolamine (PE). Phosopholipid vesicles of mitochondria-like composition (based on Henderson et al [53] for *Xenopus* mitochondria) contained (as moles percent): 48∶10∶28∶10∶4 PC/PI/PE/phosphatidylserine (PS)/cardiolipin. MGDG and DGDG were purchased from Larodan Fine Chemicals (Malmo, Sweden) and all other phospholipids were purchased from Avanti Polar Lipids Inc. (Alabaster, AL).

With the exception of Cb5, all *in vitro* synthesized proteins (OEP9, Toc33, Toc34 and SSU) were generated using the appropriate plasmid DNAs (see Materials and Methods S1) along with a T7-coupled transcription-translation system containing wheat germ extract and [^35^S]-Methionine (Perkin-Elmer NEN Radiochemicals, Waltham, MA) according to the manufacturer's instructions (Promega, Nepean, Canada). Cb5 was synthesized *in vitro* using pSP/CytoB5 plasmid DNA, SP6 polymerase (MBI Fermentas, Burlington, Canada), and RNAs translated using rabbit reticulocyte lysate in the presence of [^35^S]-Methionine as previously described [53].

Targeting of *in vitro*-translated proteins to chloroplasts was carried out as described by Smith et al [45]. Briefly, translated proteins were incubated with 50 µg of chloroplasts in HEPES-sorbitol buffer (20 mM HEPES [pH 7.5], 300 mM sorbitol), import master mix (consisting of: 50 mM HEPES-KOH, 330 mM sorbitol, 5 mM magnesium acetate, 25 mM potassium acetate), 1 mM dithiothreitol, 5 mM ATP, 1 mM GTP, 10 mM methionine and incubated at 26°C for 30 min. Following targeting, chloroplasts were reisolated by centrifugation at 2,000 *g* for 5 min at room temperature and then resuspended in either SDS-PAGE sample buffer or 100 mM Na_2_CO_3_ (pH 11.5). Chloroplasts resuspended in Na_2_CO_3_ were incubated on ice for 10 min and then centrifuged at 40,000 *g* for 30 min at 4°C using an Optima Max ultracentrifuge (Beckman Coulter Canada, Inc., Mississauga, Canada). Following centrifugation, the supernatant was isolated and subjected to trichloroacetic acid precipitation and the resulting pellet was resuspended in SDS-PAGE sample buffer. Radiolabeled proteins were analyzed by SDS-PAGE and phosphorimaging using a Bio-Rad Personal Molecular Imager FX (Bio-Rad Laboratories Ltd).

Thermolysin digestion of chloroplasts was carried out as previously described [45]. Import reactions were incubated with either 10 µg/mL thermolysin (Sigma-Aldrich Ltd.) (for import reactions with OEP9 and SSU) or 100 µg/mL thermolysin (for import reactions with Toc33 and Toc34). After a 30 min incubation on ice, EDTA was added to a final concentration of 10 mM to inactivate the protease. Thermolysin-treated chloroplasts were then repurified through a 35% (w/v) Percoll cushion and washed [45].

Pretreatment of chloroplast membranes with trypsin was carried out by incubating isolated chloroplasts with 80 µg/mL trypsin (Sigma-Aldrich Ltd.) at 25°C in the dark for 1 h as previously described [64]. The protease was then inactivated by the addition of PMSF to a final concentration of 2 mM. Trypsin-pretreated chloroplasts were then repurified, used in targeting assays, and radiolabeled proteins analyzed by SDS-PAGE/phosphoimaging as described above.

Liposome-binding assays were carried out as described previously [45]. Radiolabeled proteins were incubated with one equivalent of liposomes (40 µg) for 1 h at 24°C. Thereafter, sucrose was added to a final concentration of 1.6 M. Samples were then transferred to centrifuge tubes and sucrose gradient buffers (0.8 M and 0.25 M sucrose steps) were sequentially layered on top of the sample. After centrifugation for 18 h at 100,000 *g*, gradients were fractionated from the top into five fractions of equal volume (with the solubilized pellet as the bottom fraction) and analyzed by SDS-PAGE, using a Tris-Tricine buffer system [65] and phosphorimaging using a Storm 840 phosphorimager and allied software (GE Healthcare Life Sciences, Piscataway, NJ). All data shown from experiments with isolated chloroplasts or liposomes are representative of at least two independent experiments.

### Bioinformatics Analyses

Putative intrinsically disordered segments in OEP9 were identified using SMART (http://smart.embl-heidelberg.de/) and I-TASSER (http://zhang.bioinformatics.ku.edu/I-TASSER) protein-structure prediction programs are indicated with stippled lines. Predicted OEP9 homologues were identified by performing a WU-BLASTn (2.0) search of the Institute for Genomic Research (TIGR) plant transcript (EST) assemblies database (http://blast.jcvi.org/euk-blast/plantta_blast.cgi). Deduced amino acid sequences were then obtained from TIGR and/or GenBank (http://www.ncbi.nlm.nih.gov/) and aligned using the ClustalW algorithm (http://npsa-pbil.ibcp.fr/). The maximum likelihood phylogenetic tree represents results from neighbor-joining analysis of amino acid sequences obtained using the ClustalW2 program (http://www.ebi.ac.uk/Tools/clustalw2/index.html). Sequences used for analysis were obtained from GenBank, the *Arabidopsis* Information Resource (TAIR) (www.arabidopsis.org) and TIGR.

## Supporting Information

Figure S1 RNA and protein expression profiles of *OEP9* and selected other *Arabidopsis OEP* genes in different tissues and co-expression analysis of *OEP9*. (a) Electron (E)-northern (microarray) analyses of the *Arabidopsis* transcriptome for *OEP9* and other *OEP* genes (including those encoding individual Toc components) in various tissue types. Publicly-available *Arabidopsis* expression datasets (as of December, 2008) were explored for the chosen *Arabidopsis OEP* (*Toc*) genes using the tools available through the BioArray Resource (BAR) Expression Profiler (http://bar.utoronto.ca/) [66]. Output from the AtGenExpress_Plus extended tissue series microarray datasets [67] were formatted into a heat map using the DataMetaFormatter tool as hosted at the BAR website. Expression patterns in different tissues were expressed as averages of replicate log-transformed values normalized to the averages of the appropriate controls. Red coloring represents the highest levels of expression, as indicated by the scale. Different tissue types are indicated at the top of each heat map. Note that E-northern data (or co-expression data in [d]) for the putative *OEP9* paralogue (At1g80890) was not available since this gene is not present on the ATH1 whole genome chip. (b) Summarized is relative abundance of specific tryptic peptides representing various OEP9 and other OEPs (including several Toc components and the putative OEP9 paralogue [At1g80890] referred to here and in [c] as ‘OEP9-like’) in various tissue types. Results shown are based on data available (as of May, 2009) in the *Arabidopsis* peptide proteome TAIR7 database at the Pep2Pro (Peptide to Proteome) website (http://www.AtProteome.ethz.ch/) [68]. Quantitative values for the proteins were normalized and formatted as heat maps using the DataMetaFormatter tool as hosted at the BAR website. As indicated by the scale, red coloring represents higher levels of expression and orange or yellow coloring represents lower or no levels of expression, respectively. Different tissue types are indicated at the top of the heat map. (c) Summarized is relative abundance of massively parallel specific signature sequences (MPSSs) representing the transcript levels of *OEP9* and other *OEPs* genes (including those encoding specific Toc components) in various tissue types. Results shown are based on data available (as of December, 2008) at the *Arabidopsis* MPSS Plus website (http://mpss.udel.edu/at/) [69]. MPSS values were normalized and formatted as heat maps using the DataMetaFormatter tool as hosted at the BAR website. As indicated by the scale, red coloring represents higher levels of expression and orange or yellow coloring represents lower or no levels of expression, respectively. Different tissue types are indicated at the top of the heat map. (d) Co-expression network analysis of *OEP9.* Multiple-gene co-expression analysis was carried out using the ATTED-II (*Arabidopsis thaliana* trans-factor and *cis*-element prediction database) co-expression gene search program (http://atted.jp/) (version 5.2) [70] based on OEP9 (At1g16000) as the ‘guide gene’ and publicly-available *Arabidopsis* microarray expression datasets (as of May, 2009). Selected linkages between OEP9 (shaded circle) and other genes with a correlation coefficient of r>0.65 are indicated in the network by connecting lines, but the length of lines and distances between circles are valueless. Construction of this OEP9-guide-gene co-expression network was based on the guidelines described in Aoki et al [71] and Usadel et al [72]. Similar results were obtained using several other public databases of *Arabidopsis* gene co-expression from various experimental conditions including the PRIMe Correlated Gene Search (http://prime.psc.riken.jp/) and *Arabidopsis* Co-expression Data Mining (http://www.arabidopsis.leeds.ac.uk/act/) tools (data not shown).
(0.55 MB TIF)

Figure S2 Intracellular localization, topology and membrane insertion of OEP9. CLSM or epi-(immuno)fluorescence micrographs of either (a) BY-2 cells biolistically bombarded with empty plasmid vector DNA (pRTL2) or plasmid DNA encoding myc-OEP9, (b) *Arabidopsis* suspension-cultured cells co-transformed with myc-OEP9 and OEP7-GFP, (c) *Arabidopsis* epidermal leaf cells (from plants 30 days after sowing) co-transformed with GFP-OEP9 and Tic40-RFP, (d) BY-2 cells co-transformed with non-epitope-tagged OEP9 and myc-Toc33, or (e) BY-2 cells transformed with (non-tagged) OEP9 alone. Note that in (a) no (epi)fluorescence signal attributable to myc immunostaining is detected in representative mock (pRTL2 empty vector alone) transformed cells or when anti-myc IgGs were omitted during immunostaining of cells bombarded with DNA encoding myc-OEP9; however, both sets of representative cells in (a) display immunofluorescence attributable to the endogenous plastid enzyme NAGK. In (b) and (c), hatched boxes represent the portion of the cells shown at higher magnification in the panels or insets to the right. Solid arrowheads in (b) indicate examples of the torus structures in containing both myc-OEP9 and OEP7-GFP; the open arrowhead in (b) indicates an example of a torus structure containing OEP7-GFP, but not myc-OEP9. Solid arrowheads in (d) indicate examples of colocalization of OEP9 and myc-Toc33. Also shown for the OEP9 and myc-Toc33 co-transformed cell in (d) and GFP-OEP9 and Tic40-RFP co-transformed cell in (c) is the corresponding differential interference contrast (DIC) images. In (e) OEP9-transformed cells were differentially permeabilized with either Triton X-100 or digitonin, and then incubated with antibodies raised against either the OEP9 C-terminal sequence [refer to Figure 1a] or α-tubulin. Bars = 10 µm. (f) Insertion of non-epitope-tagged OEP9 into chloroplasts *in vitro*. Isolated *Arabidopsis* chloroplasts were incubated with *in vitro* synthesized OEP9 translation product (TP) then resuspended with Na_2_CO_3_ or incubated with thermolysin (Th). Addition of Na_2_CO_3_ or Th to the reaction mixtures is indicated as (+), omission as (−). Equivalent amounts of each Na_2_CO_3_- or mock-extracted or Th-treated chloroplast membrane sample were subjected to SDS-PAGE/phosphoimaging. On the other hand, approximately 1/40^th^ of the amount of TP that was incubated with isolated chloroplasts (lanes 2–5) was loaded in lane 1 (TP). The migration in the gel of full-length OEP9 is indicated by the solid arrowhead, whereas the resulting Th-protected fragment for this protein is indicated with open arrowhead. Note that, depending on the Th assay, the Th-protected OEP9 (and myc-OEP9) fragments observed after SDS-PAGE were sometimes diffuse (cf. lane 5 here, lane 5 in Figure 3, as well as lanes 3 and 5 in Figure S6), a feature that has been reported also for OEP14 [73] and thus is likely a general feature of low molecular weight OEPs.
(1.28 MB TIF)

Figure S3 Intracellular localization of OEP7-GFP in BY-2 cells. CLSM micrographs of cells transformed with OEP7-GFP and immunostained with antibodies against either NAGK (top row) or E1β. Hatched boxes represent the portion of the cells shown at higher magnification in the panels to the right. Solid arrowheads indicate examples of the torus fluorescent structures containing OEP7-GFP delineating the spherical structures attributable to either endogenous plastid stroma-localized NAGK or endogenous mitochondrial matrix-localized E1β. The open arrowhead indicates an example of a torus fluorescent structure that contains OEP7-GFP, but does not enclose a spherical structure containing NAGK. Bars = 10 µm.
(0.85 MB TIF)

Figure S4 Localization of myc-OEPΔCTS in BY-2 cells. Epi-(immuno)fluorescence micrographs of cells transformed either with (a) myc-OEPΔCTS or (b) co-transformed with myc-OEP9ΔCTS and GFP-OEP7. Each micrograph is labeled at the top left with the name of the expressed (fusion) protein or in (a) the endogenous organellar protein in the corresponding same cell including: mitochondrial E1β; peroxisomal catalase; and the Golgi-localized reversibly glycosylated protein (RGP). Hatched boxes represent the portion of the cells shown at higher magnification in the panels to the right. Note that in (a) the punctate structures containing expressed myc-OEP9ΔCTS do not colocalize with the punctate structures containing endogenous E1β, catalase or RGP; open arrowheads indicate examples of non-colocalization. Note also in (b) that at least some of the punctate structures (solid arrowheads) containing expressed myc-OEP9ΔCTS also contain co-expressed GFP-OEP7. Bar = 10 µm.
(0.59 MB TIF)

Figure S5 AKR2A does not mediate the nuclear relocalization of OEP7-GFP, mitochondrial Cb5 or myc-OEP9ΔCTS. Epi-(immuno)fluorescence micrographs of BY-2 cells (co-)transformed with either (a) OEP7-GFP, (b) myc-Cb5 or myc-Cb5-HA, or (c) myc-OEP9ΔCTS and NLS-RFP or NLS-RFP-AKR2A. Each micrograph is labeled at the top left with the name of either the (co-)expressed fusion protein. Also shown in (a) and (c) is the corresponding differential interference contrast (DIC) image of the OEP7-GFP or myc-OEP9ΔCTS and NLS-RFP-AKR2A co-transformed cells. Note that in (b) addition of the hemagluttinin [HA] epitope tag to the C terminus of myc-Cb5 (myc-Cb5-HA) disrupts its mitochondrial targeting information, resulting in this modified protein being mislocalized to the cytosol in BY-2 cells. Note also in (b) that myc-Cb5 and myc-Cb5-HA localize to mitochondria and cytosol, respectively, and not to the nucleus in cells co-expressing NLS-RFP-AKR2A or NLS-RFP (cf. cells expressing myc-Cb5 alone [Figure 4c]. Likewise in (a) and (c), NLS-RFP-AKR2A is not capable of mislocalizing OEP7-GFP or myc-OEP9ΔCTS to the nucleus (cf. cells either co-transformed with GFP-OEP7 and NLS-RFP-AKR2A [Figure 6c], OEP9-GFP and NLS-RFP-AKR2A [Figure 6d], or transformed with myc-OEP9ΔCTS alone [Figure 4c and S5a]). Bars = 10 µm.
(0.48 MB TIF)

Figure S6 Topology of myc-OEP9 in *ppi1* and *ppi3* chloroplasts *in vitro*. Chloroplasts isolated from *ppi1* or *ppi3* mutant *Arabidopsis* plants were incubated with *in vitro* synthesized myc-tagged OEP9 then resuspended with (+) or without (−) thermolysin (Th). Equivalent amounts of each Th-treated chloroplast membrane sample were subjected to SDS-PAGE/phosphoimaging. The migration in the gel of full-length myc-OEP9 (lanes 1, 2 and 4) is indicated by the solid arrowhead, whereas the resulting Th-protected fragment(s) for this protein (lanes 3 and 5) is indicated with an open arrowhead. Note that the Th-protected myc-OEP9 fragments observed here (lanes 3 and 5) are diffuse likely because, as mentioned above (refer to legend for Figure S2), this is a general feature commonly observed for low molecular weight OEPs after Th treatment [73].
(0.07 MB TIF)

Materials and Methods S1 (0.06 MB DOC)

Table S1 List of synthetic oligonucleotide primers used in the construction of plasmids.
(0.07 MB DOC)
