# Supplementary figures and images for: Distinct Pathways Mediate the Sorting of Tail-Anchored Proteins to the Plastid Outer Envelope
Source: PLoS One. 2010 Apr 14;5(4):e10098. doi: 10.1371/journal.pone.0010098 (PMC2854689; doi:10.1371/journal.pone.0010098)

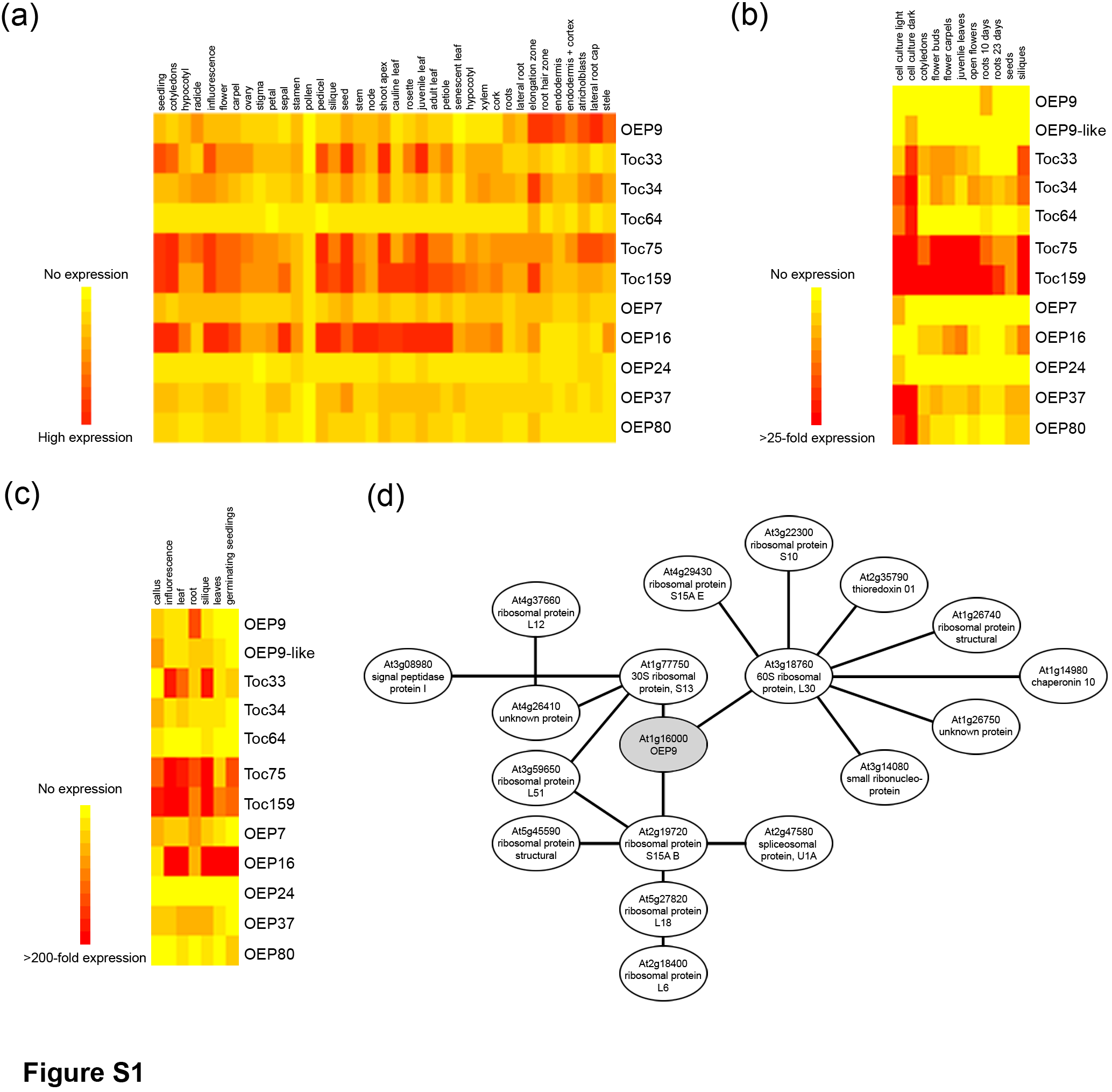

Supplement: Figure S1 — RNA and protein expression profiles of OEP9 and selected other Arabidopsis OEP genes in different tissues and co-expression analysis of OEP9. (a) Electron (E)-northern (microarray) analyses of the Arabidopsis transcriptome for OEP9 and other OEP genes (including those encoding individual Toc components) in various tissue types. Publicly-available Arabidopsis expression datasets (as of December, 2008) were explored for the chosen Arabidopsis OEP (Toc) genes using the tools available through the BioArray Resource (BAR) Expression Profiler (http://bar.utoronto.ca/) [66]. Output from the AtGenExpress_Plus extended tissue series microarray datasets [67] were formatted into a heat map using the DataMetaFormatter tool as hosted at the BAR website. Expression patterns in different tissues were expressed as averages of replicate log-transformed values normalized to the averages of the appropriate controls. Red coloring represents the highest levels of expression, as indicated by the scale. Different tissue types are indicated at the top of each heat map. Note that E-northern data (or co-expression data in [d]) for the putative OEP9 paralogue (At1g80890) was not available since this gene is not present on the ATH1 whole genome chip. (b) Summarized is relative abundance of specific tryptic peptides representing various OEP9 and other OEPs (including several Toc components and the putative OEP9 paralogue [At1g80890] referred to here and in [c] as ‘OEP9-like’) in various tissue types. Results shown are based on data available (as of May, 2009) in the Arabidopsis peptide proteome TAIR7 database at the Pep2Pro (Peptide to Proteome) website (http://www.AtProteome.ethz.ch/) [68]. Quantitative values for the proteins were normalized and formatted as heat maps using the DataMetaFormatter tool as hosted at the BAR website. As indicated by the scale, red coloring represents higher levels of expression and orange or yellow coloring represents lower or no levels of expression, respectivel [file pone.0010098.s001.tif]

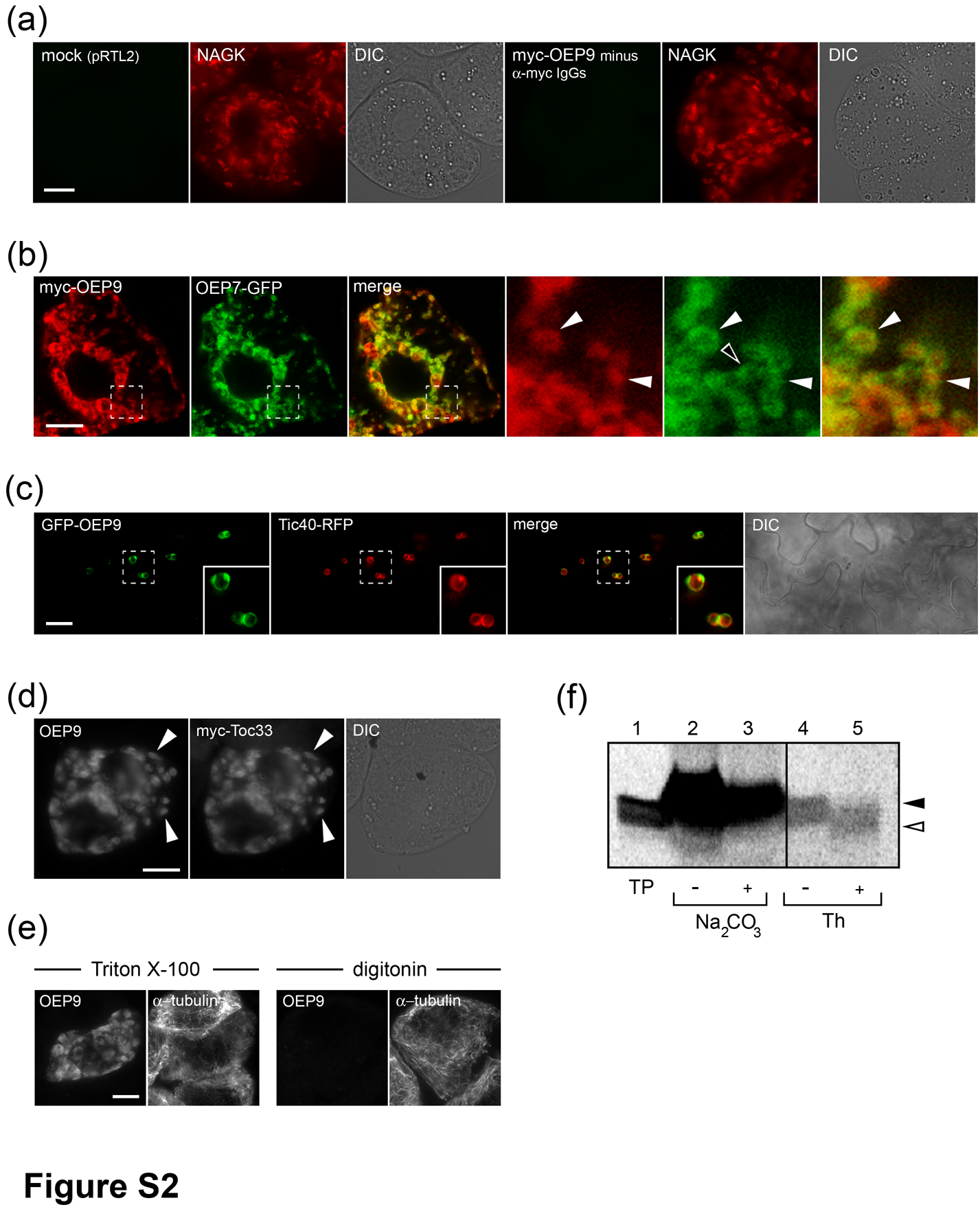

Supplement: Figure S2 — Intracellular localization, topology and membrane insertion of OEP9. CLSM or epi-(immuno)fluorescence micrographs of either (a) BY-2 cells biolistically bombarded with empty plasmid vector DNA (pRTL2) or plasmid DNA encoding myc-OEP9, (b) Arabidopsis suspension-cultured cells co-transformed with myc-OEP9 and OEP7-GFP, (c) Arabidopsis epidermal leaf cells (from plants 30 days after sowing) co-transformed with GFP-OEP9 and Tic40-RFP, (d) BY-2 cells co-transformed with non-epitope-tagged OEP9 and myc-Toc33, or (e) BY-2 cells transformed with (non-tagged) OEP9 alone. Note that in (a) no (epi)fluorescence signal attributable to myc immunostaining is detected in representative mock (pRTL2 empty vector alone) transformed cells or when anti-myc IgGs were omitted during immunostaining of cells bombarded with DNA encoding myc-OEP9; however, both sets of representative cells in (a) display immunofluorescence attributable to the endogenous plastid enzyme NAGK. In (b) and (c), hatched boxes represent the portion of the cells shown at higher magnification in the panels or insets to the right. Solid arrowheads in (b) indicate examples of the torus structures in containing both myc-OEP9 and OEP7-GFP; the open arrowhead in (b) indicates an example of a torus structure containing OEP7-GFP, but not myc-OEP9. Solid arrowheads in (d) indicate examples of colocalization of OEP9 and myc-Toc33. Also shown for the OEP9 and myc-Toc33 co-transformed cell in (d) and GFP-OEP9 and Tic40-RFP co-transformed cell in (c) is the corresponding differential interference contrast (DIC) images. In (e) OEP9-transformed cells were differentially permeabilized with either Triton X-100 or digitonin, and then incubated with antibodies raised against either the OEP9 C-terminal sequence [refer to Figure 1a] or α-tubulin. Bars = 10 µm. (f) Insertion of non-epitope-tagged OEP9 into chloroplasts in vitro. Isolated Arabidopsis chloroplasts were incubated with in vitro synthesized OEP9 translation product (TP) then [file pone.0010098.s002.tif]

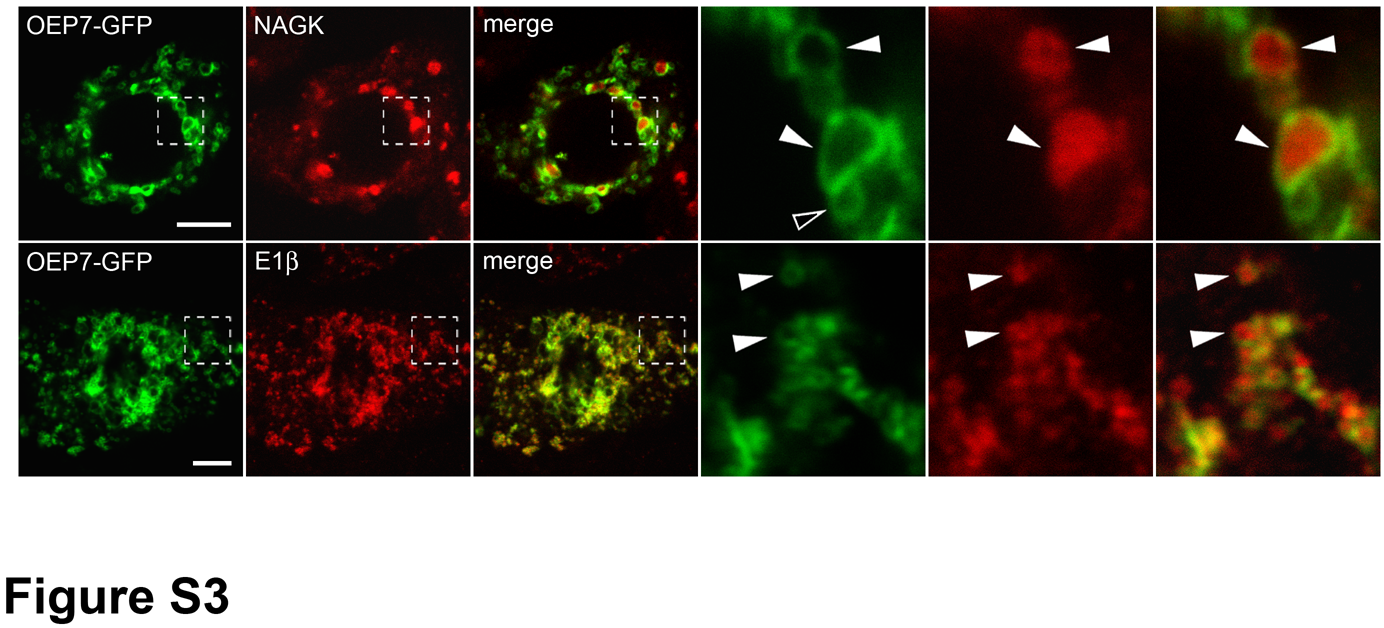

Supplement: Figure S3 — Intracellular localization of OEP7-GFP in BY-2 cells. CLSM micrographs of cells transformed with OEP7-GFP and immunostained with antibodies against either NAGK (top row) or E1β. Hatched boxes represent the portion of the cells shown at higher magnification in the panels to the right. Solid arrowheads indicate examples of the torus fluorescent structures containing OEP7-GFP delineating the spherical structures attributable to either endogenous plastid stroma-localized NAGK or endogenous mitochondrial matrix-localized E1β. The open arrowhead indicates an example of a torus fluorescent structure that contains OEP7-GFP, but does not enclose a spherical structure containing NAGK. Bars = 10 µm. (0.85 MB TIF) [file pone.0010098.s003.tif]

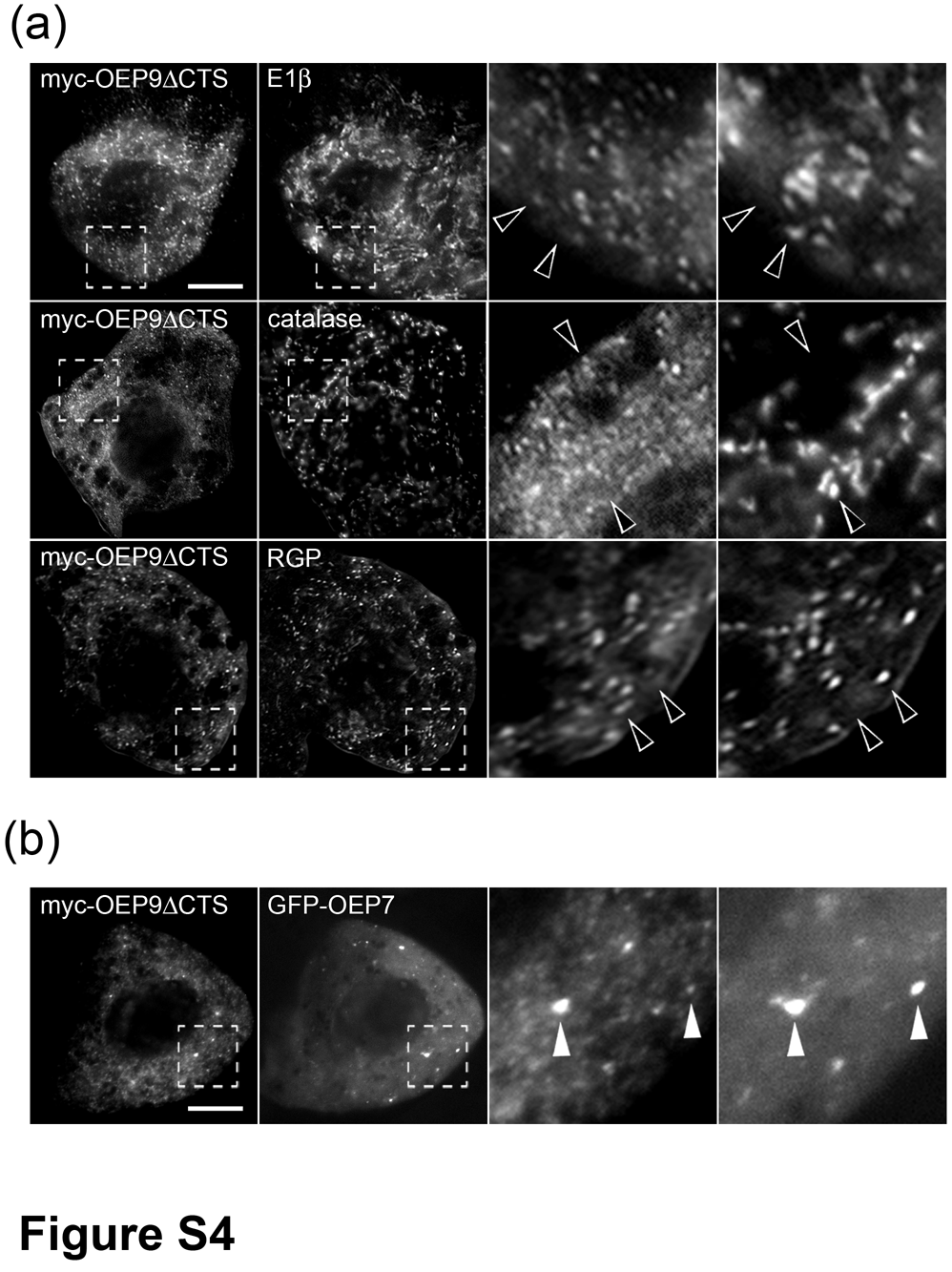

Supplement: Figure S4 — Localization of myc-OEPΔCTS in BY-2 cells. Epi-(immuno)fluorescence micrographs of cells transformed either with (a) myc-OEPΔCTS or (b) co-transformed with myc-OEP9ΔCTS and GFP-OEP7. Each micrograph is labeled at the top left with the name of the expressed (fusion) protein or in (a) the endogenous organellar protein in the corresponding same cell including: mitochondrial E1β; peroxisomal catalase; and the Golgi-localized reversibly glycosylated protein (RGP). Hatched boxes represent the portion of the cells shown at higher magnification in the panels to the right. Note that in (a) the punctate structures containing expressed myc-OEP9ΔCTS do not colocalize with the punctate structures containing endogenous E1β, catalase or RGP; open arrowheads indicate examples of non-colocalization. Note also in (b) that at least some of the punctate structures (solid arrowheads) containing expressed myc-OEP9ΔCTS also contain co-expressed GFP-OEP7. Bar = 10 µm. (0.59 MB TIF) [file pone.0010098.s004.tif]

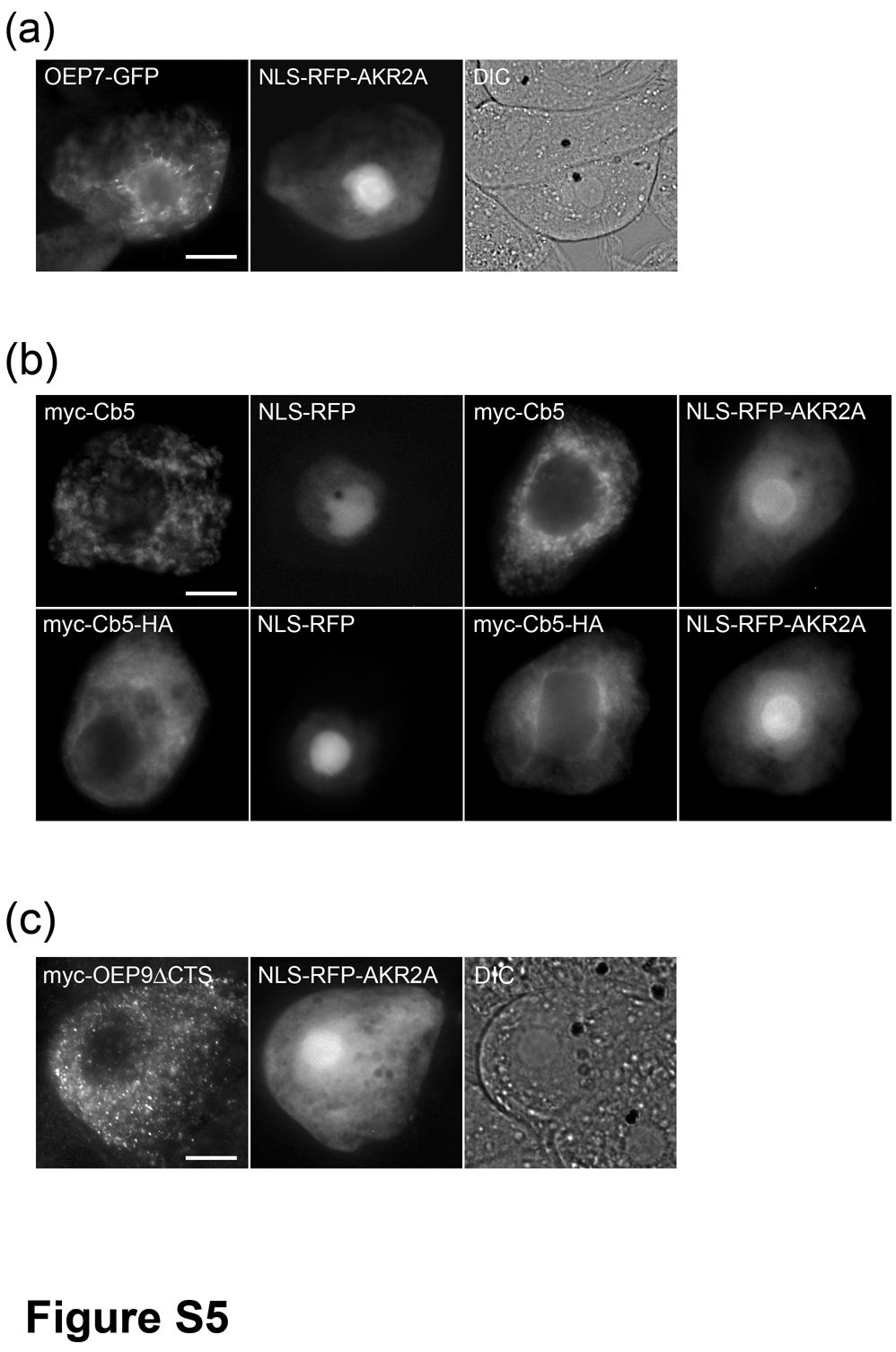

Supplement: Figure S5 — AKR2A does not mediate the nuclear relocalization of OEP7-GFP, mitochondrial Cb5 or myc-OEP9ΔCTS. Epi-(immuno)fluorescence micrographs of BY-2 cells (co-)transformed with either (a) OEP7-GFP, (b) myc-Cb5 or myc-Cb5-HA, or (c) myc-OEP9ΔCTS and NLS-RFP or NLS-RFP-AKR2A. Each micrograph is labeled at the top left with the name of either the (co-)expressed fusion protein. Also shown in (a) and (c) is the corresponding differential interference contrast (DIC) image of the OEP7-GFP or myc-OEP9ΔCTS and NLS-RFP-AKR2A co-transformed cells. Note that in (b) addition of the hemagluttinin [HA] epitope tag to the C terminus of myc-Cb5 (myc-Cb5-HA) disrupts its mitochondrial targeting information, resulting in this modified protein being mislocalized to the cytosol in BY-2 cells. Note also in (b) that myc-Cb5 and myc-Cb5-HA localize to mitochondria and cytosol, respectively, and not to the nucleus in cells co-expressing NLS-RFP-AKR2A or NLS-RFP (cf. cells expressing myc-Cb5 alone [Figure 4c]. Likewise in (a) and (c), NLS-RFP-AKR2A is not capable of mislocalizing OEP7-GFP or myc-OEP9ΔCTS to the nucleus (cf. cells either co-transformed with GFP-OEP7 and NLS-RFP-AKR2A [Figure 6c], OEP9-GFP and NLS-RFP-AKR2A [Figure 6d], or transformed with myc-OEP9ΔCTS alone [Figure 4c and S5a]). Bars = 10 µm. (0.48 MB TIF) [file pone.0010098.s005.tif]

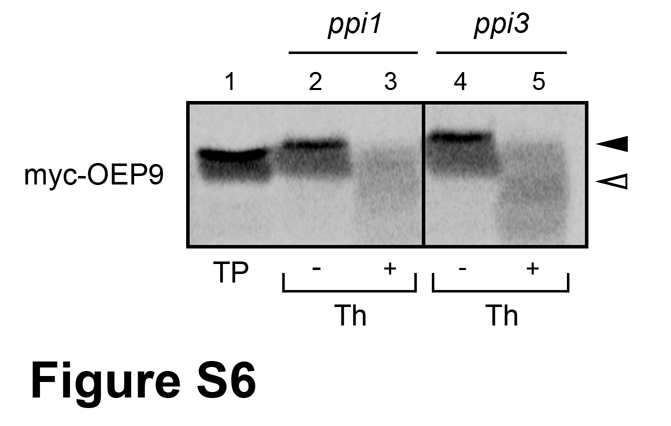

Supplement: Figure S6 — Topology of myc-OEP9 in ppi1 and ppi3 chloroplasts in vitro. Chloroplasts isolated from ppi1 or ppi3 mutant Arabidopsis plants were incubated with in vitro synthesized myc-tagged OEP9 then resuspended with (+) or without (−) thermolysin (Th). Equivalent amounts of each Th-treated chloroplast membrane sample were subjected to SDS-PAGE/phosphoimaging. The migration in the gel of full-length myc-OEP9 (lanes 1, 2 and 4) is indicated by the solid arrowhead, whereas the resulting Th-protected fragment(s) for this protein (lanes 3 and 5) is indicated with an open arrowhead. Note that the Th-protected myc-OEP9 fragments observed here (lanes 3 and 5) are diffuse likely because, as mentioned above (refer to legend for Figure S2), this is a general feature commonly observed for low molecular weight OEPs after Th treatment [73]. (0.07 MB TIF) [file pone.0010098.s006.tif]
