# Supplementary material for: Distinct Pathways Mediate the Sorting of Tail-Anchored Proteins to the Plastid Outer Envelope
Source: PLoS One. 2010 Apr 14;5(4):e10098. doi: 10.1371/journal.pone.0010098 (PMC2854689; doi:10.1371/journal.pone.0010098)
Supplement: Materials and Methods S1 — (0.06 MB DOC) [file pone.0010098.s007.doc]

# Materials and Methods S1

#

***Plasmid constructions***

A full-length cDNA (clone U50546) encoding *Arabidopsis* *thaliana* OEP9 (At1g16000) (NCBI Accession No. NP_563987) was obtained from the *Arabidopsis* Biological Resource Centre (ABRC, Columbus, OH) and plasmids containing full-length cDNAs encoding *Arabidopsis* Toc33 (pPZP221-Toc33) (At1g02280) (NCBI Accession No. NP_001117215) or Toc34 (pPZP221-Toc34) (At5g05000) (NCBI Accession No. NP_974732) were provided by P. Jarvis (University of Leicester) (Jarvis et al., 1998).

pRTL2/myc-OEP9 encoding an N-terminal myc-epitope-tagged version of OEP9 was constructed by initially amplifying (via the polymerase chain reaction [PCR]) the open reading frame (ORF) of OEP9 (with exception of its initiation methionine) using pUNI/U50546 as template DNA. The PCR also included forward and reverse oligonucleotide primers (Fp87 and Rp84; Table S1), the former introducing an *Bam*HI site immediately 5’ of the second codon in the ORF of OEP9. PCR products were gel purified and ligated into pCR2.1 TOPO (Invitrogen) and the resulting plasmid (pCR2.1 TOPO/OEP9) was digested with *Bam*HI and *Xba*I (the latter being located within the pCR2/1 TOPO multiple cloning site [MCS]) and ligated into *Bam*HI-*Xba*I-digested pRTL2/myc-BX, yielding pRTL2/myc-OEP9. Similarly, pRTL2/myc-Toc33 and pRTL2/myc-Toc34, encoding N-terminal myc-tagged versions of Toc33 and Toc34, respectively, were constructed by amplifying (via PCR) the Toc33 and Toc34 ORFs using the appropriate template DNA (pPZP221-Toc33 and pPZP221-Toc34, respectively) and forward and reverse primers (Fp135 and Rp136; Fp137 and Rp138). Both sets of primers introduced an *Xba*I site immediately 5’ of the second codon in Toc33 or Toc34, as well as an *Nhe*I site immediately 3’ of their stop codons. PCR products were subcloned into pCR2.1 TOPO (yielding pCR2.1 TOPO/Toc33 and TOPO/Toc34) and the *Xba*I-*Nhe*I fragments from these plasmids were then ligated into and the *Xba*I-*Nhe*I-digested pRTL2-myc-MCS. Both pRTL2/myc-BX and pRTL2/myc-MCS (Lingard et al., 2008) are modified versions of the plant expression vector pRTL2∆N/S (Lee et al., 1997), containing the cauliflower mosaic 35S promoter and sequences encoding an initiation methionine, glycine linkers, and the myc epitope tag (underlined, MGEQKLISEEDLG-; [74]), followed by either in-frame *BamH*I and *Xba*I sites (pRTL2/myc-BX) or an MCS (pRLT2/myc-MCS).

To construct pRTL2/GFP-Toc33, encoding Toc33 fused to the C terminus of a monomeric version of the green fluorescent protein (GFP) (i.e., the leucine at position 221 in the GFP ORF replaced with a lysine; [75]), the *Xba*I-*Nhe*I fragment from pRTL2/myc-Toc33 (see above) was ligated into *Xba*I-digested pRTL2/mGFP-MCS [76]. pUC18/OEP9-GFP and pUC18/Toc33-GFP, encoding monomeric GFP fused at its N terminus to full-length versions of OEP9 and Toc33, respectively, were constructed by amplifying (via PCR) the OEP9 and Toc33 ORFs using the appropriate template DNA (pUNI51/U50546 and pPZP221-Toc33, respectively) and forward and reverse primers (Fp339 and Rp340; Fp335 and Rp336). Both sets of primers introduced an *Nhe*I site immediately 5’ of the start codon in OEP9 or Toc33, as well as an *Nhe*I site immediately 5’ of their stop codon. The resulting PCR products were digested with *Nhe*I and ligated into *Nhe*I-digested pUC18/*Nhe*I-mGFP, a modified version of the plant expression plasmid pUC18/*Nhe*I-GFP [77] containing the cauliflower mosaic 35S promoter and an unique in-frame *Nhe*I site immediately 5’ of the ORF for monomeric GFP. pRTL2/GFP-OEP9 was constructed by ligating the *Bam*HI-*Xba*I fragment from pRLT2/myc-OEP9 into pRTL2/mGFP-OEP9. To construct pRTL2/OEP9, encoding full-length OEP9, sequences encoding the myc epitope sequence fused to the N terminus of OEP9 were removed (via site-directed mutagenesis) using pRTL2/myc-OEP9 as template DNA and the appropriate primers (Fp2838 and Rp2839).

Plant expression plasmids encoding modified versions of either OEP9, Toc33 or Toc34 fused to either the GFP or the myc epitope were constructed as follows. pRTL2/GFP-OEP9NTC, encoding GFP fused the OEP9 NTC (residues 16-86, including the 20 amino acid residues immediately upstream [N terminal] of the TMD [residues 16-35], the TMD [residues 36-54], and CTS [residues 55-86]; refer to Fig. 1) was generated by amplifying (via PCR) the appropriate sequences in the OEP9 ORF using pRTL2/myc-OEP9 as template DNA and primers (Fp166 and Rp167) that also introduced 5’ *Hind*III and 3’ *Eco*RI sites. The resulting PCR products were ligated into pCR2.1 TOPO, yielding pCR2.1 TOPO/OEP9NTC, followed by the *Hind*III-*EcoR*I fragment in this plasmid being subcloned into *Hind*III/*EcoR*I-digested pRTL2/mGFP-MCS. pRTL2/GFP-OEP9CTS was generated by using pRTL2/GFP-OEP9_31-86_ as template DNA to delete (via site-directed mutagenesis; Fp221 and Rp222) the sequences in OEP9 encoding residues 31 to 49, including most of the protein’s TMD, yielding a plasmid (pRTL2/GFP-OEP9CTS) encoding GFP fused to the C-terminal 50-86 residues of OEP9. pRTL2/GFP-OEP9_31-86_, encoding GFP fused to the C-terminal 55 amino acids of the OEP9, including five residues upstream (N-terminal) of the TMD (residues 31-35) plus its entire TMD and CTS (residues 36-86), was constructed by amplifying (via PCR) the appropriate sequences from pRTL2/myc-OEP9 using primers (Fp212 and Rp167) that also introduced 5’ *Hind*III and 3’ *EcoR*I sites. The resulting PCR products were ligated into pCR2.1/TOPO, yielding pCR2.1TOPO/OEP9_31-86_, and the *Hind*III-*Eco*RI fragment in this latter plasmid was then ligated into *Hind*III/*EcoR*I-digested pRTL2/mGFP-MCS. pRTL2/myc-OEP9∆NTC was generated by replacing (via site-directed mutagenesis; Fp217 and Rp218) the leucine at position -37 in the OEP9 ORF with a stop codon in pRTL2/myc-OEP9. Similarly, pRTL2/myc-OEP9∆CTS was generated by replacing (via site-directed mutagenesis; Fp205 and Rp206) the serine at position 57 in the OEP9 ORF with a stop codon in pRTL2/myc-OEP9. pRTL2/myc-OEP9_1-70_ was generated by replacing (via site-directed mutagenesis; Fp325 and Rp327) the lysine at position 72 in the OEP9 ORF with a stop codon, using pRTL2/myc-OEP9 as template DNA. pRTL2/myc-OEP9D_68_D_71_∆G, pRTL2/mycOEP9K_69_K_72_R_74_K_75_∆G, and pRLT2/myc-OEP9Y_66_M_67_A_70_∆G were all generated using site-directed mutagenesis and pRTL2/myc-OEP9 as template DNA, along with the appropriate primers to change to glycines either the aspartate residues at positions 68 and 71 (Fp341 and RP342), the lysine and arginine residues at positions 69, 72, 74 and 75 (Fp343 and Rp344), or the tyrosine, methionine and alanine residues at positions 66, 67 and 70, respectively (Fp345 and Rp346). pRTL2/myc-OEP9∆Toc33CTS was generated by replacing (via site-directed mutagenesis; Fp324 and Rp325) the OEP9 CTS (residues 55 to 86) with the Toc33 CTS (residues 282 to 297) using pRTL2/myc-OEP9 as template DNA.

pRTL2/GFP-Toc33NTC, encoding monomeric GFP fused at its C terminus to the Toc33 NTC (residues 246-297, including the 20 amino acid residues immediately upstream [N terminal] of the TMD [residues 246-266], the TMD [residues 267-282], and CTS [residues 283-297]; refer to Fig. 4a), was constructed as follows. First, the appropriate sequences in the Toc33 ORF were amplified (via PCR; Fp164 and Rp167) from pRTL2/myc-Toc33 along with 5’ *Hind*III and 3’ *Eco*RI sites. The resulting PCR products were then ligated into pCR2.1 TOPO, yielding pCR2.1 TOPO/Toc33NTC, followed by the *Hind*III-*EcoR*I fragment in this latter plasmid being subcloned into *Hind*III/*EcoR*I-digested pRTL2/mGFP-MCS. pRTL2/GFP-Toc33_141-297_ was constructed by amplifying (via PCR; Fp213 and Rp167) the sequences encoding the C-terminal 156 amino acids (residues 141-297) of the Toc33 ORF from pRTL2/myc-Toc33 along with 5’ *Hind*III and 3’ *EcoR*I sites. The resulting PCR products were digested with *Hind*III and *EcoR*I and ligated into *Hind*III/*EcoR*I-digested pRTL2/mGFP-MCS. pRTL2/GFP-Toc33NTC∆OEP9CTS encoding monomeric GFP fused to the 20 amino acid residues immediately upstream [N terminal] of the Toc33 TMD (residues 248-266), the Toc33 TMD (residues 267-281), along with the OEP9 CTS (residues 55-86) and a small portion of the OEP9 TMD (residues 50-54) was generated as follows. First an *Nhe*I site was introduced (via site-directed mutagenesis; Fp308 and Rp309) at positions 282 and 283 in the Toc33 ORF in pRTL2/GFP-Toc33NTC, yielding pRTL2/mGFP-Toc33NTCΔ*Nhe*I*.* Next, the sequences encoding residues 50-86 and stop codon in the OEP9 ORF were amplified (via PCR; Fp310 and Rp311) from pRTL2/myc-OEP9 along with 5’ and 3’ *Nhe*I sites, and then the resulting PCR products were digested with *Nhe*I and ligated into *Nhe*I-digested pRTL2/mGFP-Toc33NTCΔ*Nhe*I, yielding pRTL2/GFP-Toc33NTC∆OEP9CTS. pRTL2/myc-Toc33∆NTC was generated by replacing (via site-directed mutagenesis; Fp215 and Rp216) the lysine at position 246 in the Toc33 ORF with a stop codon in pRTL2/myc-Toc33. pRTL2/myc-Toc33_37-297_ was generated by deleting (via site-directed mutagenesis; Fp313 and Rp312) the sequences encoding the N-terminal 36 amino acids in the Toc33 ORF (i.e., the residues immediately upstream of the GTPase domain [residues 37-234, refer to Fig. 4a]) in pRTL2/myc-Toc33. pRTL2/myc-Toc33R_130_∆A was constructed by replacing (via site-directed mutagenesis; Fp318 and Rp319) the arginine at position 130 in the Toc33 ORF with an alanine in pRTL2/myc-Toc33.

pRTL2/GFP-Toc34NTC encoding monomeric GFP fused at its C terminus to the Toc34 NTC (residues 249-313, including the 20 amino acid residues immediately upstream [N terminal] of the TMD [residues 249-268], the TMD [residues 269-286], and CTS [residues 287-313]; refer to Fig. 4a), was constructed as follows. The appropriate sequences in the Toc34 ORF were amplified (via PCR; Fp165 and Rp167) from pRTL2/myc-Toc34 along with 5’ *Hind*III and 3’ *EcoR*I sites. The resulting PCR products were ligated into pCR2.1 TOPO yielding pCR2.1 TOPO/Toc34NTC, followed by the *Hind*III-*EcoR*I fragment in this latter plasmid being subcloned into *Hind*III/*EcoR*I-digested pRTL2/mGFP-MCS.

The construction of pRTL2/myc-Cb5, encoding an N-terminal myc-tagged version of tung (*Aleurites fordii*) tree Cb5 isoform D (NCBI Accession No. Ay578730) has been described previously [17]. pRTL2/myc-Cb5ΔOEP9CTS encoding a modified version of myc-Cb5 in which its CTS was replaced with the CTS from OEP9 was constructed as follows. First, sequences encoding residues 50-86 and stop codon in the OEP9 ORF, along with 5’ and 3’ *Nhe*I sites, were amplified (via PCR; Fp310 and Rp311) from pRTL2/myc-OEP9. The resulting PCR products were then digested with *Nhe*I and ligated into *Xba*I-digested pRTL2/myc-Cb5 to yield pRTL2/myc-Cb5ΔOEP9CTS. Next, sequences encoding the CTS (residues 132-134) and stop codon in Cb5 were removed (via site directed mutagenesis; Fp347 and Rp348), yielding pRTL2/myc-Cb5ΔOEP9CTS.

pRTL2/NLS-RFP-AKR2A, encoding *Arabidopsis* AKR2A fused to nuclear-localized version of the red fluorescent protein (RFP) was constructed in three steps. First, annealed (complementary) oligonucleotides (Fp22 and Rp22) encoding three repeats of the simian virus 40 (SV40) large antigen nuclear localization signal (NLS), -KKRKKV- [78], along with an initiation methionine with *Nco*I compatible ends, were ligated directly into *Nco*I-digested pRTL2/MCS-RFP, yielding pRTL2/NLS-RFP. Second, the stop codon in the RFP ORF in pRTL2/NLS-RFP was removed (via site-directed mutagenesis; Fp24 and Rp24), yielding pRTL2/NLS-RFP∆TAA. Third, sequences encoding the AKR2A ORF (NCBI Accession No. NP_849499) (At3g35450.4), along with 5’ and 3’ *Xba*I sites, were amplified (via PCR; Fp330 and Rp331) from pUNI51/U15717 (obtained from the ABRC) and the resulting PCR products were ligated into *Xba*I-digested pRTL2/NLS-RFP∆TAA, yielding pRTL2/NLS-RFP-AKR2A. pRTL2/MCS-RFP is a modified version of the plant expression vector pRTL2
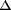
N/S that contains the ORF for a monomeric version of the Discosoma sp. RFP downstream of an MCS [76]. pRTL2/GFP-AKR2A was generated by ligating the same *Xb*aI-digested PCR products used in the construction of pRTL2/NLS-RFP-AKR2A (see above) into *Xba*I-digested pRTL2/mGFP-MCS.

pUC18/OEP7-GFP, consisting of *Arabidopsis* OEP7 (NCBI Accession No. NP_190810) fused to the N terminus of the monomeric GFP was generated by amplifying (via PCR; Fp332 and Rp333) the sequences encoding the full-length OEP7 ORF (with exception of its stop codon), along with 5’ and 3’ *Xba*I sites, from pUNI/U61692 (obtained from the ABRC). The resulting PCR products were then digested with *Xba*I and ligated into *Nhe*I-digested pUC18/*Nhe*I-mGFP, yielding pUC18/OEP7-GFP. pRTL2/GFP-OEP7 was generated by first ligating the *Xb*aI-digested PCR product used in the construction of pUC18/OEP7-GFP (see above) into *Xba*I-digested pRTL2/mGFP-MCS to yield pRLT2/mGFP-OEP7-ns. Next, sequences encoding a stop codon at the 3’ end of the OEP7 ORFP were added (via site directed mutagenesis; Fp349 and Rp350) in pRLT2/mGFP-OEP7-ns, yielding pRTL2/GFP-OEP7.

pRLT2/Tic40-RPF, consisting of the *Arabidopsis* 40 kDa component of the translocon at the inner membrane of chloroplasts (Tic40) (At5g16620) (NCBI Accession No. NP_197165) fused to the N terminus of GFP was constructed by amplifying (via PCR; Fp248 and Rp249) the sequences encoding the full-length Tic40 ORF (with exception of its stop codon), along with 5’ and 3’ *Xma*I restriction sites, from pUNI51/U17194 (obtained from the ABRC). The resulting PCR products were ligated into pCR2.1 TOPO (yielding pCR2.1 TOPO/Tic40) and the *Xma*I-*Xma*I fragment was then excised from pCR2.1 TOPO/Tic40 and ligated into *Xma*I-digested pRTL2/MCS-RFP, yielding pRTL2/Tic40-RFP.

Plasmids encoding proteins used in *in vitro* membrane insertion experiments were constructed in the following manner. pSPUTK/myc-OEP9, encoding the full-length, N-terminal-myc-tagged OEP9 was generated by ligating the *Nco*I-*Xba*I-digested fragment from pRTL2/myc-OEP9 (see above) into *Nco*I-*Xba*I-digested pSPUTK-*Bgl*II-*Nhe*I. Likewise, plasmids encoding pSPUTK/myc-Toc33 and pSPUTK/myc-Toc34 were generated by ligating the *Nco*I-*Nhe*I-digested fragments from pRTL2/myc-Toc33 and pRTL2/myc-Toc34, respectively, into *Nco*I-*Nhe*I-digested pSPUTK-*Bgl*II-*Nhe*I. pSPUTK-*Bgl*II-*Nhe*I is a version of pSPUTK-*Bgl*II [79] that contains the SP6 promoter, the high-efficiency β-globin 5’ untranslated region (UTR), a Kozak’s initiation site [80] for efficient translation in reticulocyte lyaste, and a modified MCS. Specifically, pSPUTK-*Bgl*II-*Nhe*I was generated by first digesting pSPUTK-*Bgl*II with *Bgl*II and *EcoR*I to remove the sequences corresponding to its MCS and then ligating with annealed (complementary) oligonucleotides (Fp128 and Rp129) coding for an MCS with *Bgl*II, *BamH*I, *Nco*I, *Kpn*I, *Xma*I, *Xba*I, *Nhe*I and *EcoR*I restriction sites. Thereafter, a second *Nhe*I restriction site in the plasmid (i.e., outside of the MCS) was removed (via site-directed mutagenesis; Fp150 and Rp151), yielding pSPUTK-*Bgl*II-*Nhe*I. pSPUTK/OEP9 was constructed by amplifying (via PCR; Fp2813 and Rp2814) the sequences encoding the full-length OEP9 ORF, along with 5’ *Nco*I and 3’ *Xma*I restiction sites, from pRTL2/GFP-OEP9. The resulting PCR fragments were then digested with *Nco*I and *Xma*I, gel purified and ligated into *Nco*I-*Xma*I-digested pSPUTK-*Bgl*II-*Nhe*I.

pET21A/SSU encoding the full-length *Arabidopsis* ribulose bisphosphate carboxylase small subunit 1B (SSU) (At5g38430) (NCBI Accession No. AB005248) was constructed by first amplifying (via PCR; FpAtsB*Nde*l and RpAts1B*Xho*I) the sequences encoding the full-length SSU ORF, along with 5’ *Nde*I and 3’ *Xho*I restriction sites, from *Arabidopsis thaliana* (ecytope Wassilewskija) cDNA. The resulting PCR products were then digested with *Nde*I and *Xho*I and ligated into *Nde*I-*Xho*I-digested pET21A (EMD Chemical Inc. [previously Novagen], Gibbstown, NJ) yielding pET21A/SSU. Construction of pSP/CytoB5 containing the full-length ORF of the ER isoform of rat liver Cb5 (NCBI Accession No. AF007108) has been described elsewhere [81].
