# Supplementary material for: Distinct Pathways Mediate the Sorting of Tail-Anchored Proteins to the Plastid Outer Envelope
Source: PLoS One. 2010 Apr 14;5(4):e10098. doi: 10.1371/journal.pone.0010098 (PMC2854689; doi:10.1371/journal.pone.0010098)
Supplement: Table S1 — List of synthetic oligonucleotide primers used in the construction of plasmids. (0.07 MB DOC) [file pone.0010098.s008.doc]

Table S1. List of synthetic oligonucleotide primers used in the construction of plasmids.

| Plasmid | Primers | Sequence (5' to 3') |
| --- | --- | --- |
| pRTL2/myc-OEP9 | Fp87 | CCAGAAGGAGATATAGGATCCGGAAATGAGACGAAG |
|  | Rp84 | GAGCGCTCACAATTCTCTAGAAGGCCCATGAGGCC |
|  |  |  |
| pRTL2/myc-Toc33 | Fp135 | CGGGTTTAGGAGTGTCTAGATCTCTCGTTCGTG |
|  | Rp136 | GCTCACAACAAAAGCTAGCCTTCAGAGCCTC |
|  |  |  |
| pRTL2/myc-Toc34 | Fp137 | GTAAGGATTTGTGTCCTCTAGAGCTTTGCAAACGC |
|  | Rp138 | CCAAACAAAACTCGTGCTAGCAACATCAGACCTTCG |
|  |  |  |
| pUC18/OEP9-GFP | Fp339 | GGGCCCGGCCGGCCGCTAGCATGGGAAATGAGACGAAGACC |
|  | Rp340 | GGCCGGGCCCGGCGGCTAGCCTTGTTAGCTGATGAAGACGATGAG |
|  |  |  |
| pUC18/Toc33-GFP | Fp335 | GGGCCCGGCCGGCCGCTAGCATGGGGTCTCTCGTTCGTGAATGGG |
|  | Rp336 | GGCCGGGCCCGGCGGCTAGCAAGTGGCTTTCCACTTGTCTTGATATC |
|  |  |  |
| pRTL2/OEP9 | Fp2838 | GTTGGTCTTCGTCTCATTTCCCATGGCTATCGTTCGTAAATG |
|  | Rp2839 | CATTTACGAACGATAGCCATGGGAAATGAGACGAAGACCAAC |
|  |  |  |
| pRTL2/GFP-OEP9NTC | Fp166 | AACGGTGGTCCTGCAAAGCTTGCCGGAGGCGGAGGA |
|  | Rp167 | GATAGATTTGTAGAGAGAATTCGGTGATTTTGCGGAC |
|  |  |  |
| pRTL2/GFP-OEP9CTS | Fp221 | GGATGAACTATACAAACCTTGGTATTTGATGACTCAA |
|  | Rp222 | TTGAGTCATCAAATACCAAGGTTTGTATAGTTCATCC |
|  |  |  |
| pRTL2/GFP-OEP9_31-86_ | Fp212 | GGGCCCAAGCTTGGTGGAAAGAAGCACTGCTTGG |
|  | Rp167 | GATAGATTTGTAGAGAGAATTCGGTGATTTTGCGGAC |
|  |  |  |
| pRTL2/myc-OEP9∆NTC | Fp217 | GGTGAAAAGAAGCAGGTCTAAGTCGGGATCGGAATCG |
|  | Rp218 | CGATTCCGATCCCGACTTAGACCTGCTTCTTTTCACC |
|  |  |  |
| pRTL2/myc-OEP9∆CTS | Fp205 | ATTTGATGACTCAAGGGTAAAAGCATCAATCTCACCC |
|  | Rp206 | CTTGAGTCATCAAATGGGTGAGATTGATGCTTTTACC |
|  |  |  |
| pRTL2/myc-OEP9_1-70_ | Fp326 | ACATGGACAAGGCCGATTAAGCTCGAAAAGCACGCC |
|  | Rp327 | GGCGTGCTTTTCGAGCTTAATCGGCCTTGTCCATGT |
|  |  |  |
| pRTL2/myc-OEP9_DD∆GG_ | Fp341 | CACCAAGATTACATGGGCAAGGCCGGTAAAGCTCGAAAAGCACGCC |
|  | Rp342 | GGCGTGCTTTTCGAGCTTTACCGGCCTTGCCCATGTAATCTTGGTG |
|  |  |  |
| pRTL2/myc-OEP9_KKRK∆GGGG_ | Fp343 | CCAAGATTACATGGACGGGGCCGATGGGGCTGGAGGGGCACGCCTCTCATCGTCTTC |
|  | Rp344 | GAAGACGATGAGAGGCGTGCCCCTCCAGCCCCATCGGCCCCGTCCATGTAATCTTGG |
|  |  |  |
| pRTL2/myc-OEP9_YMA∆GGG_ | Fp345 | CAATCTCACCAAGATGGCGGGGACAAGGGCGATAAAGCTCGAAAAG |
|  | Rp346 | CTTTTCGAGCTTTATCGCCCTTGTCCCCGCCATCTTGGTGAGATTG |
|  |  |  |
| pRTL2/GFP-Toc33NTC | Fp164 | GTAGCAACAAACCAGAAGCTTGCAATTCATGTAGACAAG |
|  | Rp167 | GATAGATTTGTAGAGAGAATTCGGTGATTTTGCGGAC |
|  |  |  |
| pRTL2/GFP-Toc33_141-297_ | Fp213 | GGGCGCAAGCTTGATTGCCTTGAATCATCTTAACG |
|  | Rp167 | GATAGATTTGTAGAGAGAATTCGGTGATTTTGCGGAC |
|  |  |  |
| pRTL2/myc-Toc33∆NTC | Fp215 | GCAACAAACCAGAGGTAAGCAATTCATGTAGAC |
|  | Rp216 | GTCTACATGAATTGCTTACCTCTGGTTTGTTGC |
|  |  |  |
| pRTL2/myc-Toc33_37-297_ | Fp313 | GAAGAAGATCTGTCTAGAAAGTATAAAGAAGAGGATG |
|  | Rp312 | CATCCTCTTCTTTATACTTTCTAGACAGATCTTCTTC |
|  |  |  |
| pRTL2/myc-Toc33_R∆A_ | Fp318 | CTTGCTCTATGTTGATGCTTTGGATGTGTATAGAG |
|  | Rp319 | CTCTATACACATCCAAAGCATCAACATAGAGCAAG |
|  |  |  |
| pRTL2/GFP-Toc34∆NTC | Fp165 | GAGATCTCTTTTAATGGCAAGCTTGCGATTCATGTTC |
|  | Rp167 | GATAGATTTGTAGAGAGAATTCGGTGATTTTGCGGAC |
|  |  |  |
| pRTL2/GFP-Toc33NTC∆OEP9CTS | Fp308 | ATGAAGCCATTGGTTCGAGCTAGCAAGTCCGATGTTTCG |
|  | Rp309 | CGAAACATCGGACTTGCTAGCTCGAACCAATGGCTTCAT |
|  | Fp310 | GGCCGGCCGCTAGCTCAAAGCATCAATCTCACCAAG |
|  | Rp311 | GGCCGGCCGCTAGCCTACTTGTTAGCTGATGAAGA |
|  |  |  |
| pRTL2/myc-OEP9∆Toc33CTS | Fp324 | GGAGTTCCTTGGTATTTGCAAGGACAAGTGGAAAGCCACTTTAAGAGCAATCAGAAA  TGATATCAAATGACTCAAGGGTCAAAGC |
|  | Rp325 | GCTTTGACCCTTGAGTCATTTAAAGTGGCTTTCCACTTGTCTTGATATCATTTCTGA  TTGCTCCTTGCAAATACCAAGGAACTCC |
|  |  |  |
| pRTL2/myc-Cb5∆OEP9CTS | Fp310 | GGCCGGCCGCTAGCTCAAAGCATCAATCTCACCAAG |
|  | Rp311 | GGCCGGCCGCTAGCCTACTTGTTAGCTGATGAAGA |
|  | Fp347 | GTTGGCTTCTTATACCTAACTCAAGGGTCAAAGCATCAATCTCACC |
|  | Rp348 | GGTGAGATTGATGCTTTGACCCTTGAGTTAGGTATAAGAAGCCAAC |
|  |  |  |
| pRTL2/NLS-RFP | Fp22 | CATGGGCCCGAAGAAAAAGAGGAAGGTCCCGAAAAAGAGGAAG  GTCCCGAAGAAAAAGAGGAAGGTCGC |
|  | Rp22 | CATGGCGACCTTCCTCTTTTTCTTCGGGACCTTCCTCTTTTTT  CTTCGGGACCTTCCTCTTTTTCTTCGGGCC |
|  |  |  |
| pRTL2/NLS-RFP∆TAA | Fp24 | GCCGCCACTCCACCGGCGCCTATAGAGTCCGCAAAAATCACC |
|  | Rp24 | GGTGATTTTTGCGGACTCTATAGGCGCCGGTGGAGTGGCGGC |
|  |  |  |
| pRTL2/NLS-RFP-AKR2A | Fp333a | CGCCCCGCTCTAGAATGGCTTCCAATTCGGAGAAAAATCC |
|  | Rp334 | GGCCGGCCTCTAGATCAAAGGAAAGCATCCTTCTCAAGC |
|  |  |  |
| pUC18/OEP7-GFP | Fp332 | GGCCCGGGGCTAGCATGGGAAAAACTTCGGGAGCG |
|  | Rp333 | GGCCCGGGGCTAGCCAAACCCTCTTTGGATGTGG |
|  |  |  |
| pRTL2/GFP-OEP7 | Fp332 | GGCCCGGGGCTAGCATGGGAAAAACTTCGGGAGCG |
|  | Rp333 | GGCCCGGGGCTAGCCAAACCCTCTTTGGATGTGG |
|  | Fp349 | AAGAGGGTTTGTAATCTAGCGTCCGC |
|  | Rp350 | GCGGACGCTAGATTACAAACCCTCTT |
|  |  |  |
| pRTL2/Tic40-RFP | Fp248 | GCCGCGCCCGGGATGGAGAACCTTACCC |
|  | Rp249 | GCCGCGCCCGGGACCCGTCATTCCTGGG |
|  |  |  |
| pSPUTK-*Bgl*II-*Nhe*I | Fp128 | GATCTGGATCCCCATGGGGTACCCCCGGGTCTAGAGCTAGCG |
|  | Rp129 | AATCCGCTAGCTCTAGACCCGGGGGTACCCCATGGGGATCCA |
|  | Fp150 | GAGGATCTGGCTAGGGATGACCCTGCT |
|  | Rp151 | AGCAGGGTCATCCCTAGCCATATCCTC |
|  |  |  |
| pSPUTK/OEP9 | Fp2813 | GGCCGGCCATGGGAAATGAGACGAAGACC |
|  | Rp2814 | GGCCGGCCCGGGCTACTTGTTAGCTGATGAAGAC |
|  |  |  |
| pET21d-pSSU | Ats1B*Nde*IFp | GAAGAACATATGGCTTCCTCTATGCTC |
|  | Ats1B*Xho*IRp | CAGAACTCGAGTTAAGCATCAGTGAAG |
